# A Female-Biased Chemosensory Protein PxutCSP19 in the Antennae of *Papilio xuthus* Tuned to Host Volatiles and Insecticides

**DOI:** 10.3390/insects15070501

**Published:** 2024-07-05

**Authors:** Ningna Yin, Dan Shen, Yinlan Liang, Pengfei Wang, Yonghe Li, Naiyong Liu

**Affiliations:** 1 Key Laboratory of Forest Disaster Warning and Control of Yunnan Province, Southwest Forestry University, Kunming 650224, China; yinningna@swfu.edu.cn (N.Y.); sd18687816301@swfu.edu.cn (D.S.); 16606930423@swfu.edu.cn (Y.L.); 202212103006@swfu.edu.cn (P.W.); 2 Key Laboratory of National Forestry and Grassland Administration on Biodiversity Conservation in Southwest China, Southwest Forestry University, Kunming 650224, China; 3 College of Plant Protection, Yunnan Agricultural University, Kunming 650201, China

**Keywords:** swallowtail butterfly, chemosensory protein, host volatile, insecticide, extended N-terminus

## Abstract

**Simple Summary:**

The swallowtail butterfly *Papilio xuthus* is a specialized herbivorous insect, with its larvae solely utilizing the Rutaceae plants as hosts. The host seeking and orientation of this butterfly mainly rely on chemosensory-related genes expressed in the antennae, including chemosensory proteins (CSPs). Here, we characterized the putative roles of a female-antenna-biased PxutCSP19 in *P. xuthus* associated with the perception of host volatiles and the sequestering of insecticides. This study examined the key functions of PxutCSP19 in the interactions of six host-derived odorants and eight insecticides. Notably, an extended N-terminus of PxutCSP19 did not significantly affect the binding specificities of this protein to ligands with high affinities. Our findings provide insight into the binding mechanisms of PxutCSP19 in *P. xuthus* to host volatiles and insecticides.

**Abstract:**

Chemosensory protein (CSP) genes significantly enriched in the female antennae are potential molecular candidates for mediating female oviposition behaviors. In this study, we presented the interaction mechanisms of a female-antenna-biased PxutCSP19 in *Papilio xuthus* to 47 host volatiles, four biopesticides and 24 synthetic insecticides. Using a bioinformatics-based homology search, 22 genes orthologous to PxutCSP19 were identified from 22 other *Papilio* butterflies with high sequence identities to each other (73.20~98.72%). Multiple alignment analyses revealed a particularly extended N-terminus of *Papilio* CSP19s (an average of 154 residues) compared to insects’ typical CSPs (approximately 120 residues). The expression profiles indicated that PxutCSP19 was significantly enriched in the female antennae, with a 31.81-fold difference relative to the male antennae. In ligand-binding assays, PxutCSP19 could strongly bind six host odorants with high affinities, ranging from dissociation constant (K_i_) values of 20.44 ± 0.64 μM to 22.71 ± 0.73 μM. Notably, this protein was tuned to a monoterpenoid alcohol, linalool, which generally existed in the Rutaceae plants and elicited electrophysiological and behavioral activities of the swallowtail butterfly. On the other hand, PxutCSP19 was also capable of binding eight insecticides with stronger binding abilities (K_i_ < 12 μM) compared to host odorants. When an extended N-terminal region of PxutCSP19 was truncated into two different proteins, they did not significantly affect the binding of PxutCSP19 to ligands with high affinities, suggesting that this extended N-terminal sequences were not involved in the specificity of ligand recognition. Altogether, our study sheds light on the putative roles of PxutCSP19 enriched in the female antennae of *P. xuthus* in the perception of host volatiles and the sequestering of insecticides, and it complements the knowledge of butterfly CSPs in olfaction and insecticide resistance.

## 1. Introduction

Moths and butterflies constitute two sister groups of the Lepidoptera, with approximately 160,000 recognized species, as moth species account for nearly 90% of all lepidopteran insects [1,2,3]. Their larvae are typical plant-feeding insects that are distinguished into three feeding habits based on the host range, i.e., monophagy, oligophagy and polyphagy. Compared with polyphagous herbivores, monophagous and oligophagous feeders possibly need to develop a more sensitive and sophisticated chemosensory system related to gustation and olfaction, particularly for female butterflies responsible for the seeking of oviposition hosts. The Papilionidae family is a representative of Papilionoidea, also comprising six other families, and constitutes 570 described species, accounting for over 3% of all butterfly members. The larvae of most Papilionid species feed on a limited number of host plants, such as the genus *Papilio* mainly eating the Rutaceae plants [4,5,6]. Thus, in the stenophagous *Papilio* butterflies, it is of paramount importance for the seeking and orientation of the right host plants as food resources and oviposition sites, as well as the assessment of food quality and safety by utilizing their chemosensory systems [7].

Tissue-enriched or sex-biased olfactory genes in chemosensory organs are able to govern specific behaviors of female and/or male insects. The antenna is the most important olfactory organ that guides the searching of host plants in herbivorous insects [8]. Chemosensory proteins (CSPs) enriched in antennae have been found to be determinants of recognizing the hosts of feeding and oviposition. These cases have been observed in insect species, for example, CchiCSP1 in *Callosobruchus chinensis* to mung bean volatiles [9], AlinCSP1, AlinCSP2 and AlinCSP3 in *Adelphocoris lineolatus* with cotton- and alfalfa-produced odorants [10], DabiCSP1 in *Dioryctria abietella* to pine secondary metabolites [11], as well as CmedCSP33 in *Cnaphalocrocis medinalis* [12], CsupCSP1, CsupCSP2 and CsupCSP3 in *Chilo suppressalis* [13], NlugCSP8 and NlugCSP10 in *Nilaparvata lugens* [14,15] and SfurCSP5 in *Sogatella furcifera* [16] to rice volatiles.

Similar to odorant-binding proteins (OBPs), sex-biased CSPs in the antennae also serve as male- or female-specific olfactory-related behaviors. In *Athetis lepigone*, a male-biased AlepCSP2 highly expressed in the antennae mediated the perception of the female sex pheromones *Z*7-dodecenyl acetate and *Z*9-tetradecadienyl acetate [17]. Such roles in sex pheromone detection were also observed in *Mamestra brassicae*, *Sesamia inferens* and *Plutella xylostella*, in which MbraCAPA6, SinfCSP19 and PxylCSP11 could interact strongly with the respective sex pheromones of female moths [18,19,20]. By contrast, female-antenna-biased CSPs, in most cases, are correlated with the specific olfactory events, primarily referring to the detection of oviposition sites. In *Apolygus lucorum*, AlucCSP1 was identified as a significantly antenna-enriched gene in female bugs responding to cotton-derived secondary metabolites [21]. With similar findings, female-biased CSPs enriched in the antennae of several species have been suggested to participate in female oviposition behaviors, comprising MsepCSP5 and MsepCSP8 in *Mythimna separata* [22,23], CforCSP6 in *Cylas formicarius* [24], AmalCSP5 in *Agrilus mali* [25] and GmmCSP2 in *Glossina morsitans morsitans* [26].

In comparison to other insects, butterfly CSPs expressed in the antennae have received relatively less attention, particularly for their roles in the interactions of host volatiles and insecticides. Moreover, to the best of our knowledge, no CSP functional investigations in butterflies have been reported to date. Here, we addressed the putative roles of PxutCSP19 in a Rutaceae-feeding swallowtail butterfly, *Papilio xuthus*, associated with host recognition and insecticide sequestering. Given the female-biased expression of PxutCSP19 in the antennae, we hypothesize that this protein possibly participates in the determination of oviposition sites via utilizing host-derived volatiles. In the process of searching oviposition hosts and achieving other life activities, on the other hand, *P. xuthus* adults may contact toxic substances (like insecticides) where antennal CSPs may sequester these toxic compounds so as to protect them from reaching their target proteins [27]. To test the hypotheses, we first harvested the PxutCSP19 protein by a combination of a prokaryotic expression system and affinity chromatography. Furthermore, its ligand-binding properties to 47 host volatiles, four biopesticides and 24 synthetic insecticides were determined. Considering that PxutCSP19 possessed a longer N-terminus (more than 35 residues) compared to typical CSPs (approximately 120 amino acids), we truncated this extended N-terminal sequence into two different regions and constructed two truncated proteins. Afterwards, we determined the effects of this extended N-terminus of PxutCSP19 on the specificity of ligand recognition. Our findings suggest that this female-antenna-biased PxutCSP19 in the swallowtail butterfly is a candidate molecular target for recognizing host odorants and binding insecticides.

## 2. Materials and Methods

### 2.1. Butterflies and Dissection of Body Parts

*P. xuthus* eggs were collected from an insect-breeding farm in Suqian City, Jiangsu Province, China. They were kept in a controlled rearing room at 25 ± 1 °C and 60 ± 10% relative humidity in a 14:10 h light/dark photoperiod. After egg hatching, the larvae were raised on the leaves of the Dahongpao Sichuan pepper *Piperis dahongpao* until the body part collections, comprising the antennae, maxillary palps, labra, spinnerets, heads (removing the antennae), stink glands, silk glands, tracheae, fat bodies, ventral nerves, foreguts, midguts, hindguts, Malpighian tubules and epidermis from the 5th instar larvae. Briefly, each body part was isolated and cleaned in phosphate-buffered saline (PBS, pH 7.4), with 10~100 larvae as one biological template and at least 4 sets for each body part. During the period of body part dissection, 1.5 mL Eppendorf tubes were kept on dry ice.

For the collection of adult body parts, the larvae were reared until pupation, followed by adult emergence. Newly emerged individuals were separated by sex according to the external genitalia and maintained in different cages with a 10% sugar solution and fermented fruit juices. The body part collections were conducted by hand-dissecting the antennae, proboscises, heads (excluding the antennae and proboscises), thoraxes, abdomens (excluding the reproductive systems), legs and wings, as well as reproductive organs (i.e., accessory glands, ejaculatory ducts, seminal vesicles and testes of males; accessory glands, ovaries, bursa copulatrix and spermatheca connecting spermathecal glands of females). Four biological pools were prepared with 5~50 adults for each template. All the collected body parts were immediately frozen in liquid nitrogen and stored at –80 °C.

### 2.2. RNA Isolation and cDNA Synthesis

The total RNAs of the collected body parts were extracted using TRIzol reagent (Invitrogen Life Technologies, Carlsbad, CA, USA), following the manufacturer’s instructions. In brief, these body parts were ground using glass homogenizers (Solarbio Life Sciences, Beijing, China) by adding 1 mL TRIzol. After centrifugation, the supernatant was collected for further RNA purification. Finally, the RNA samples were dissolved in 30~50 μL nuclease-free water. A NanoDrop 1000 Spectrophotometer (Thermo Fisher Scientific, San Jose, CA, USA) and agarose gel electrophoresis (1%, *w*/*v*) were used to measure the quality and concentration of the RNA samples. A PrimeScript™ reagent Kit with gDNA Eraser (TaKaRa, Dalian, Liaoning, China) was employed to the prepared cDNA templates from 1 μg of total RNA, including a DNase digestion step at 42 °C for 2 min.

### 2.3. Gene Identification

We first identified PxutCSP19 (GenBank accession number: XP_013162723.1) in the antennal transcriptome of female *P. xuthus* (SRA accession number: DRX276979) via a blast-based homology search implemented in Geneious R10.1.3 (https://www.geneious.com/, accessed on 5 May 2024). Afterwards, a search of the National Center for Biotechnology Information (NCBI) Genome database with the term “*Papilio*” yielded 22 genome assemblies derived from 22 *Papilio* butterflies (https://www.ncbi.nlm.nih.gov/genome/?term=papilio, accessed on 15 May 2024). Thus, we also conducted the identification of PxutCSP19 orthologs in the 22 genome assemblies. On the other hand, putative PxutCSP19 orthologs were found in five other lepidopteran species, including *Kallima inachus* [28], *Heliconius melpomene* [29], *Manduca sexta* [30], *Helicoverpa armigera* [31] and *Achelura yunnanensis* [32]. Of these, except for AyunCSP44 derived from the transcriptomes of *A. yunnanensis*, the CSPs in the other four species were annotated from their respective genomes. Specifically, when genes were identified as partial sequences, the corresponding transcriptome data were retrieved from the NCBI Sequence Read Archive (SRA) database (https://www.ncbi.nlm.nih.gov/sra, accessed on 15 May 2024) to extend the missing sequences if available. Detailed information on the genome assemblies of the 27 lepidopteran species is provided in Table S1.

### 2.4. Sequence and Structure Analysis

For *Papilio* CSP19 genes, we conducted the following analyses: (1) prediction of signal peptides with SignalP–6.0 Server [33]; (2) exon–intron structural analyses using GeneWise according to the GT–AG rule between exon and intron boundaries, including the numbers, sizes and insertion sites of introns [34]; (3) generation of gene structure graphics with Exon-Intron Graphic Maker (http://wormweb.org/exonintron, accessed on 10 June 2024); (4) multiple alignments of protein sequences using MAFFT v7.490 [35] and editing of the resulting alignments with Jalview v2.8 [36]; (5) prediction of the secondary structure based on the crystal structure of SgreCSP4 in *Schistocerca gregaria* (PDB ID: 2GVS) [37] and (6) chromosomal localization by mapping the CSP sequences onto the reference genomes.

### 2.5. Expression Profiling Analysis of PxutCSP19 in P. xuthus

To investigate the expression profiles of PxutCSP19 in various body parts of *P. xuthus*, PCR strategies were employed to determine its body parts’ distribution and sex-biased transcription. We initially examined the distribution of PxutCSP19 in 29 body parts and eight reproductive organs using reverse transcription PCR (RT–PCR) assays. The quality-control of the templates was performed using ribosomal protein S4 (PxutRPS4) [38]. Reactions were run on a TAdanced 96 G Detection System (Analytik Jena AG, Jena, Germany), with a 25 μL mixture for each tube encompassing 2.5 μL of 10 × PCR buffer (Mg^2+^), 2 μL of dNTP mixture (each 2.5 mM), 2 μL of cDNA templates (~30 ng), 1.5 μL of forward or reverse primers (10 μM) and 15.5 μL of nuclease-free water. The procedure was set at 90 °C for 3 min, 35 cycles of 94 °C for 30 s, 58 °C for 30 s and 72 °C for 40 s, and a final extension for 10 min at 72 °C. Based on the RT–PCR results, quantitative real-time PCR (qPCR) was employed to measure the relative expression levels of PxutCSP19 in the detectable body parts. The run was carried out on a qTOWER 2.2 Real Time PCR Thermal Cycler (Analytik Jena AG, Jena, Germany), in which each well contained a total of 20 μL mixture with three technical replicates and three biological pools for each body part. A Bestar SybrGreen qPCR Mastermix (DBI^®®^ Bioscience, Ludwigshafen, Germany) was used to amplify the products, together with the cDNA templates and primers. The normalized expression levels of PxutCSP19 were calculated using its cycle threshold (CT) values relative to the CT values of PxutRPL8 [38,39] in a Q-Gene package [40,41]. The primers for the expression profiles are listed in Table S2.

### 2.6. Protein Expression and Purification

The open reading frame (ORF) of PxutCSP19 was cloned with a PrimeSTAR^®®^ Max DNA Polymerase (TaKaRa, Liaoning, Dalian, China) and gene-specific primers containing restriction enzyme sites (BamH I and Xho I) (Table S2). The PCR products were detected on a 1.5% (*w*/*v*) agarose gel, and the expected bands were cut and purified using a HiPure Gel Pure DNA Mini Kit (Magen, Guangzhou, China). Next, BamH I and Xho I were used to digest the PxutCSP19 products and pET-30a (+) plasmids, followed by purification. A T4 DNA ligase (Thermo Fisher Scientific, Waltham, MA, USA) was applied to the construction of the recombinant plasmids pET-30a (+)/PxutCSP19. After being confirmed by sequencing, the plasmids were transformed into BL21 (DE3) competent cells so as to express recombinant proteins with 1.0 mM isopropyl β-D-thiogalactoside (IPTG). Because this recombinant protein pET-30a (+)/PxutCSP19 was insoluble, we applied a protein denaturation–renaturation approach to purify PxutCSP19 with an affinity chromatography technique, in which the denaturation buffer included 8 M urea and 1.0 mM β-mercaptoethanol. Because the recombinant proteins possessed a His-tag sequence at the N-terminus, we next used enterokinase (GenScript, Nanjing, China) to remove this tag, followed by re-purification.

### 2.7. Host Volatiles and Insecticides

The host volatiles of *P. xuthus* were reported and collected in previous studies, with a total of 47 chemicals consisting of 18 alkenes, 12 alcohols, 11 aldehydes, three esters and three other odorants derived from the Rutaceae plants [42,43,44,45]. Given the importance of CSPs to insect resistance [27,46], we also included 24 synthetic insecticides belonging to 11 chemical classes and four biopesticides that have been reported to be applied in the control of lepidopteran pests and showed strong interactions with insect CSPs [27,46]. As a female-biased PxutCSP19 in the antennae, it was suggested that this gene was possibly involved in the detection of male sex pheromones in the swallowtail butterfly. However, the sex pheromones of male *P. xuthus* remain unknown. Thus, in the present study, we did not measure the binding of PxutCSP19 to these semiochemicals. All the chemicals, including N-phenyl-1-naphthylamine (1-NPN) and methanol (HPLC grade, 99.9%), were obtained from Sigma-Aldrich (St. Louis, MO, USA) and Aladdin (Shanghai, China). In the binding assays, the chemicals were dissolved and diluted in methanol at an appropriate concentration.

### 2.8. Fluorescence Competitive Binding Assays

In order to investigate the ligand-binding properties of PxutCSP19 to host odorants and insecticides, we first asked whether this fluorescent probe 1-NPN was available for the binding of PxutCSP19. To calculate the binding ability of PxutCSP19 to 1-NPN, we titrated different final concentrations of 1-NPN (2~20 μM) into Tris-HCl buffer (pH 7.4) containing 2 μM PxutCSP19. The binding constant (K_1-NPN_) of 1-NPN to this protein was determined with GraphPad Prism 7 (GraphPad Software Inc., San Diego, CA), as previously described [47]. The solution was excited at 337 nm and the emission peak was monitored at 414 nm on an RF-5301PC spectrofluorometer (Shimadzu, Kyoto, Japan) using a quartz micro-cuvette filled with a 100 μL mixture, with 5 nm excitation and emission slit widths. If the host odorant molecules could displace more than 40% of the 1-NPN fluorescence from the binding cavity of PxutCSP19, the compounds would be continuously added up to 36 μM with triplicates. For the binding experiments, each compound would be added into the PxutCSP19/1-NPN (each 2 μM) complex with final concentrations of 2~20 μM. Between the wildtype and two truncated PxutCSP19s, six host volatiles (ocimene, terpinolene, 1-octanol, linalool, nerol and syringaldehyde) and eight insecticides (chlorfluazuron, hexaflumuron, chlorpyrifos, phoxim, indoxacarb, monosultap, chlorfenapyr and rotenone) that showed available dissociation constant (K_i_) values were further compared and analyzed. We used the K_i_ values to compare the binding affinities of various ligands with the equation: K_i_ = [IC_50_]/(1 + [1-NPN]/K_1-NPN_), where IC_50_ represents the ligand concentrations displacing one half of the initial protein/1-NPN fluorescence, and [1-NPN] is the free concentration of 1-NPN at 2 μM.

### 2.9. Construction of Two Truncated PxutCSP19s

To determine the effects of an extended N-terminal sequence (approximately 35 amino acids) on the binding specificity of PxutCSP19 to ligands, we next constructed two truncated PxutCSP19s. Firstly, the front 17 amino acids (i.e., EITIGGIERTMSPGVKS, defined as PxutCSP19-T1) and the front 35 amino acids (i.e., EITIGGIERTMSPGVKSMGYKIIYGEDDIAEVNEV, defined as PxutCSP19-T2) of the N-terminal region without the signal peptide were truncated, respectively. Secondly, the two truncated ORFs (PxutCSP19-T1: 360 bp and PxutCSP19-T2: 306 bp) of PxutCSP19s were separately amplified using RT–PCR with gene-specific primers (Table S2). After that, the amplified products were sub-cloned into pET-30a (+), i.e., pET-30a (+)/PxutCSP19-T1 and pET-30a (+)/PxutCSP19-T2. Using the above-described methods for the expression and purification of proteins, we obtained two truncated proteins. Furthermore, the binding characteristics of the truncated PxutCSP19s to ligands with high affinities were investigated, representing six host odorants and eight insecticides.

### 2.10. Statistical Analyses

The error bars in this study denote the standard error of the mean (SEM). The gene expression levels among the body parts were compared using one-way analysis of variance (ANOVA), followed by Fisher’s LSD test, in IBM SPSS Statistics 21.0 (SPSS Inc., Chicago, IL, USA). A significant difference was set at *p* < 0.05 with different lowercase letters. For the comparison of the binding affinities between the wildtype and truncated PxutCSP19s, a two-tailed unpaired heteroscedastic Student’s *t*-test was employed to determine the statistical significance (*p* < 0.05), as Levene’s test of homogeneity demonstrated that the variances between the binding affinities (1/K_i_) of the wildtype and truncated proteins were unequal in IBM SPSS Statistics 21.0. The normality of the data was checked using the one-sample Kolmogorov–Smirnov test in GraphPad Prism 7.

## 3. Results

### 3.1. The Identification of CSP19 Orthologs in Papilio Butterflies

We first identified PxutCSP19 from the antennal transcriptome of female *P. xuthus* with the SRA accession number of DRX276979. In the available genomes of the other 22 *Papilio* species, our bioinformatics-based homologous analyses also led to the yields of 22 PxutCSP19 orthologs as a singleton in each *Papilio* butterfly. All the identified genes encoded complete ORFs with sizes of 151~157 amino acids, except for PslaCSP19 (126 amino acids) in *Papilio slateri* and PhelCSP19 (134 amino acids) in *Papilio helenus*, which missed approximately 25 and 23 residues in the middle of the two sequences, respectively. At the N-terminal regions, these CSP19 proteins shared signal peptides of 16~17 amino acids (Table 1).

**Figure 1:**
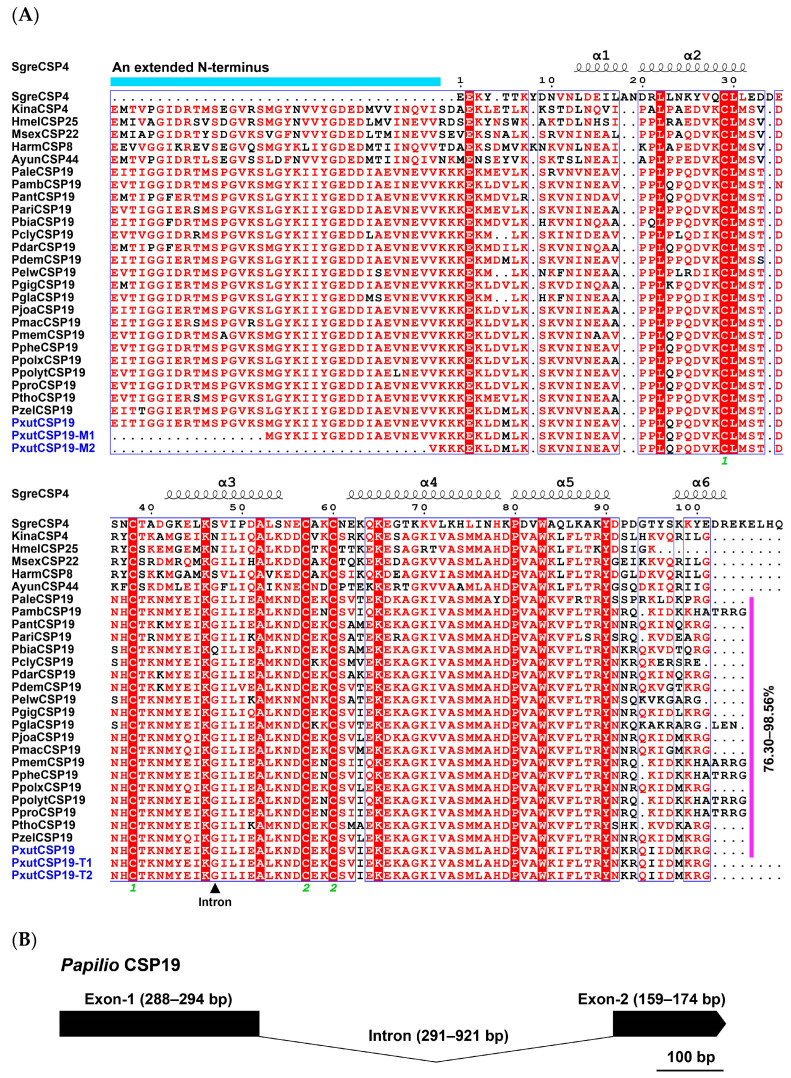
Multiple alignment of amino acid sequences based on 21 *Papilio* CSP19s and their orthologs in five other lepidopteran species. (**A**) The alignment of amino acid sequences of PxutCSP19 and its orthologs. Using the crystal structure of SgreCSP4 in *Schistocerca gregaria* (PDB ID: 2GVS) as a template [37], locations of six α-helixes for *Papilio* CSP19s and their orthologs were predicted. Compared to SgreCSP4, *Papilio* CSP19s and their orthologs possessed an extended N-terminus with around 35 residues. Amino acid identities among 21 *Papilio* CSP19s (removing signal peptides) are indicated with 76.30~98.56%. Four conserved cysteines that form two disulfide bonds are numbered 1 to 2. A triangle represents the insertion sites of introns within a codon, representing a phase-1 intron that is located the first and the second bases of a codon. Two truncated PxutCSP19s (PxutCSP19-T1 and PxutCSP19-T2) were included in this alignment. (**B**) Gene structure of *Papilio* CSP19s. The lengths of the exons (Exon-1 and Exon-2) and introns are indicated on the top of the structure.

Among the 21 full-length CSP19s, they exhibited an average of 82.91% protein sequence identity. Two CSP19 orthologs between *Papilio clytia* and *Papilio memnon* showed 73.20% amino acid identity, while the highest pairwise identities (98.72%) were observed between PpheCSP19 in *Papilio phestus* and PambCSP19 in *Papilio ambrax* or PpolytCSP19 in *Papilio polytes*. In comparison, a moderate amino acid identity (50~60%) of the CSP19 orthologs was observed between *Papilio* and five other lepidopteran species (*A. yunnanensis*, *H. armigera*, *K. inachus*, *H. melpomene* and *M. sexta*). Interestingly, the NCBI blast hits and sequence alignment analyses revealed that PxutCSP19 and its orthologs harbored an extended N-terminus (more than 35 amino acids) compared to insects’ typical CSPs (approximately 120 amino acids) (Figure 1A).

We next analyzed the gene structure of the 21 CSP19s by mapping the sequences onto their respective genomes, except for two partial sequences. These orthologous genes, at least those CSPs with known structures, shared highly conserved exon and intron structures, i.e., conserved exon/intron numbers as well as intron phases and insertion sites. By comparison, the transcriptional orientation of the genes, intron lengths and exon-2 sizes were more diverse (Figure 1B and Table 1).

### 3.2. Tissue- and Sex-Specific Expression of PxutCSP19 in P. xuthus

**Figure 2:**
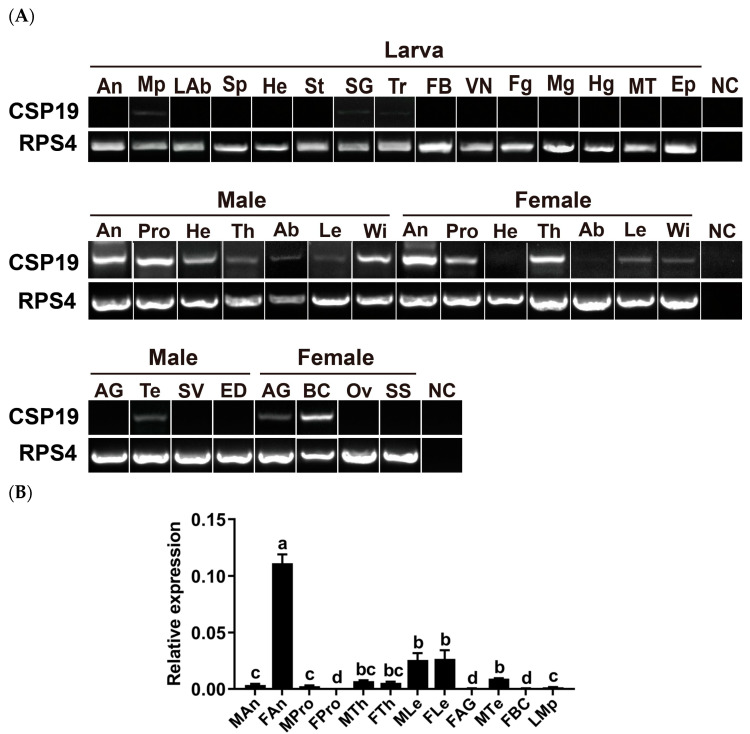
Expression profile of PxutCSP19 in body parts of *P. xuthus*. (**A**) RT–PCR analysis of PxutCSP19 in 29 body parts and eight reproductive organs. An, antennae; Mp, maxillary palps; LAb, labra; Sp, spinnerets; He, heads; St, stink glands; SG, silk glands; Tr, tracheae; FB, fat bodies; VN, ventral nerves; Fg, foreguts; Mg, midguts; Hg, hindguts; MT, Malpighian tubules; Ep, epidermis; Pro, proboscises; Th, thoraxes; Ab, abdomens; Le, legs; Wi, wings; AG, accessory glands; Te, testes; SV, seminal vesicles; ED, ejaculatory ducts; BC, bursa copulatrix; Ov, ovaries; SS, spermathecae and spermathecal glands; NC, negative control using sterile water as the template. The quality of the cDNA templates was checked using PxutRPS4. (**B**) qPCR analysis of PxutCSP19 in nine body parts and three reproductive organs. Based on the RT–PCR results, chemosensory and non-chemosensory body parts of larvae and adults were selected, including larval maxillary palps (LMp) as well as antennae, proboscises, legs and thoraxes of both sexes. Additionally, three reproductive organs (female accessory glands, female bursa copulatrix and male testes) with the detectable expression were also included. Statistical significance is denoted with different lowercase letters (ANOVA, Fisher’s LSD test, *p* < 0.05). Relative expression levels of PxutCSP19 were normalized relative to PxutRPL8.

To determine the expression profiles of PxutCSP19, we comprehensively surveyed its distribution in 29 body parts of larval and adult butterflies as well as eight reproductive organs. RT–PCR analyses revealed that this gene was primarily expressed in the adult body parts, resulting in a robust amplification in the antennae and proboscises of both sexes, male wings, female thoraxes and female bursa copulatrix. At the larval stage, it appeared that this gene had no detectable expression, with the exceptions of the maxillary palps and silk glands showing extremely weak bands (Figure 2A and Supplementary File S1). Using qPCR assays, we further examined the relative expression levels of PxutCSP19 in body parts. In line with the RT–PCR results, PxutCSP19 had a significantly higher transcriptional level in the female antennae, which was 31.81-fold higher compared to the male antennae (Figure 2B).

### 3.3. Bacterial Expression and Purification of PxutCSP19

**Figure 3:**
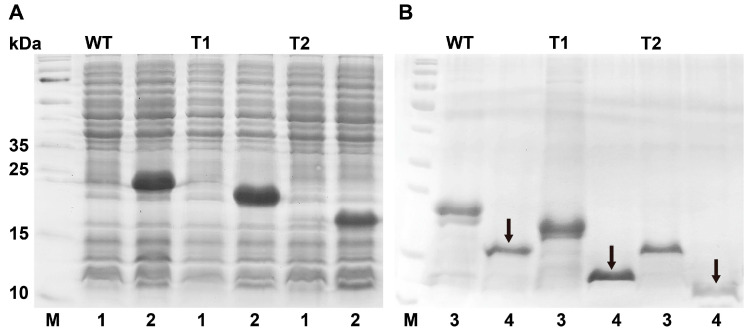
Bacterial expression and purification of PxutCSP19 in *P. xuthus*. (**A**) The expression of pET-30a (+)/PxutCSP19s. Recombinant proteins were induced by IPTG, including the wildtype (WT) and truncated (T1 and T2) pET-30a (+)/PxutCSP19s. Line 1 and 2, pET-30a (+)/PxutCSP19s without or with IPTG induction, respectively. (**B**) The purification of the wildtype and truncated PxutCSP19s. Line 3 and 4, purified PxutCSP19 proteins with or without his-tags, respectively. Arrows indicate target proteins of the wildtype and truncated PxutCSP19s. M, protein molecular weight marker.

To investigate the putative roles of PxutCSP19 in host recognition, we first expressed and purified the protein. After 1.0 mM IPTG induction, an apparently crude band was observed with a size of 20.82 kDa (Figure 3A). Next, this recombinant protein was obtained via the affinity chromatography technique, followed by re-purification with the digestion of enterokinase. A single band was detected at the expected position of 15.39 kDa, as indicated by an arrow in the SDS-PAGE analyses (Figure 3B and Supplementary File S2).

### 3.4. Binding Properties of PxutCSP19 to Host Volatiles

In the interactions of PxutCSP19 and the fluorescent probe 1-NPN, a strong binding was observed, representing a K_1-NPN_ value of 2.65 ± 0.35 μM. Moreover, the binding of this protein to 1-NPN had a good linear relationship, as revealed by the Scatchard plot, suggesting a single binding site (Figure S1A). Herein, we used 1-NPN as the fluorescent probe to characterize the binding properties of PxutCSP19 to host odorants. Out of the 47 tested host-derived compounds, the majority of them only displaced a few 1-NPN molecules from the binding pocket of PxutCSP19, ranging from 5.65% 1-NPN fluorescence for α-cypermethrin to 46.40% for γ-terpinene at 20 μM. Unexpectedly, PxutCSP19 had no binding with two alkenes (β-caryophyllene and β-elemene). With these observations, we further examined the binding of 10 host odorants (ocimene, γ-terpinene, α-terpinene, terpinolene, 1-octanol, linalool, nerol, 4-terpineol, α-terpineol and syringaldehyde) to PxutCSP19, in which these compounds had an over 40% fluorescent displacement rate at 20 μM. By extending a range of ligand concentrations (up to 36 μM), it was noted that six (ocimene, terpinolene, 1-octanol, linalool, nerol and syringaldehyde) out of the ten ligands exhibited strong interactions with this protein, representing K_i_ values of 20.44 ± 0.64~22.71 ± 0.73 μM. However, the remaining four compounds (i.e., γ-terpinene, α-terpinene, 4-terpineol and α-terpineol) could not displace more 1-NPN molecules (less than 50% 1-NPN fluorescence at 36 μM) from the binding pocket of PxutCSP19 when constantly adding odorant molecules into the PxutCSP19/1-NPN mixture (Figure S1B and Table 2).

### 3.5. Binding Properties of PxutCSP19 to Insecticides

To characterize the putative roles of PxutCSP19 in insecticide resistance, we additionally determined the binding characteristics of this protein to 24 synthetic insecticides and four biopesticides belonging to different chemical classes. In comparison to plant odorants, PxutCSP19 was capable of sequestering eight insecticides at a relatively lower concentration of chemicals (IC_50_ < 20 μM). In particular, two broad-spectrum organophosphorus insecticides, phoxim (K_i_ = 1.73 ± 0.08 μM) and chlorpyrifos (K_i_ = 3.64 ± 0.11 μM), were identified as the best ligands. Apart from that, a botanical insecticide rotenone was also strongly bound by PxutCSP19, with a K_i_ value of 6.03 ± 0.50 μM. Notably, approximately half of the remaining 20 ligands (9/20 insecticides) were able to displace a moderate number of 1-NPN molecules from the binding pocket of PxutCSP19, ranging from 41.04% to 48.54% 1-NPN fluorescence at 20 μM (Figure S1B and Table 2).

### 3.6. Effects of an Extended N-Terminus on the Binding Specificity of PxutCSP19 to Ligands

**Figure 4:**
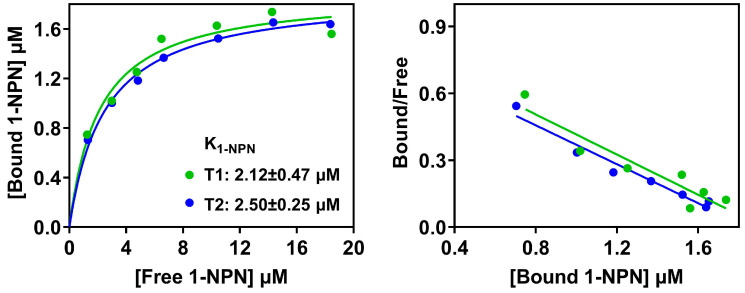
Binding of two truncated PxutCSP19 proteins to 1-NPN and relative Scatchard plots.

As mentioned above, PxutCSP19 and its orthologs possessed an extended N-terminus with about 35 more residues compared to typical CSPs. We asked whether this additional region at the N-terminus affected the interactions of PxutCSP19 and ligands. Accordingly, we constructed two variants by truncating 17 (PxutCSP19–T1) and 35 (PxutCSP19–T2) amino acids, respectively. Similar to the wildtype protein, two truncated PxutCSP19s were expressed in this bacterial system, with the expected sizes (20.82 kDa) and high yields (Figure 3A). After purification, the proteins were harvested with single bands at the positions of 13.63 kDa for PxutCSP19–T1 and 11.59 kDa for PxutCSP19–T2 (Figure 3B). We next measured the binding of the two truncated proteins to 1-NPN, revealing their strong interactions (PxutCSP19–T1, K_1-NPN_ = 2.12 ± 0.47 μM; PxutCSP19–T2, K_1-NPN_ = 2.50 ± 0.25 μM) and good linear relationships, similar to the wildtype protein (Figure 4).

**Figure 5:**
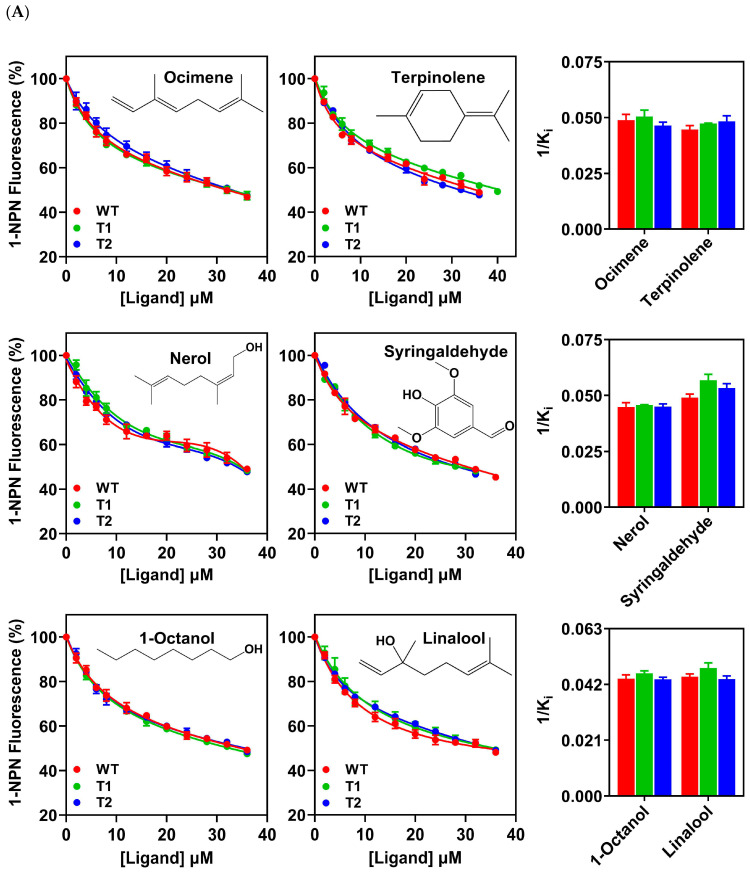

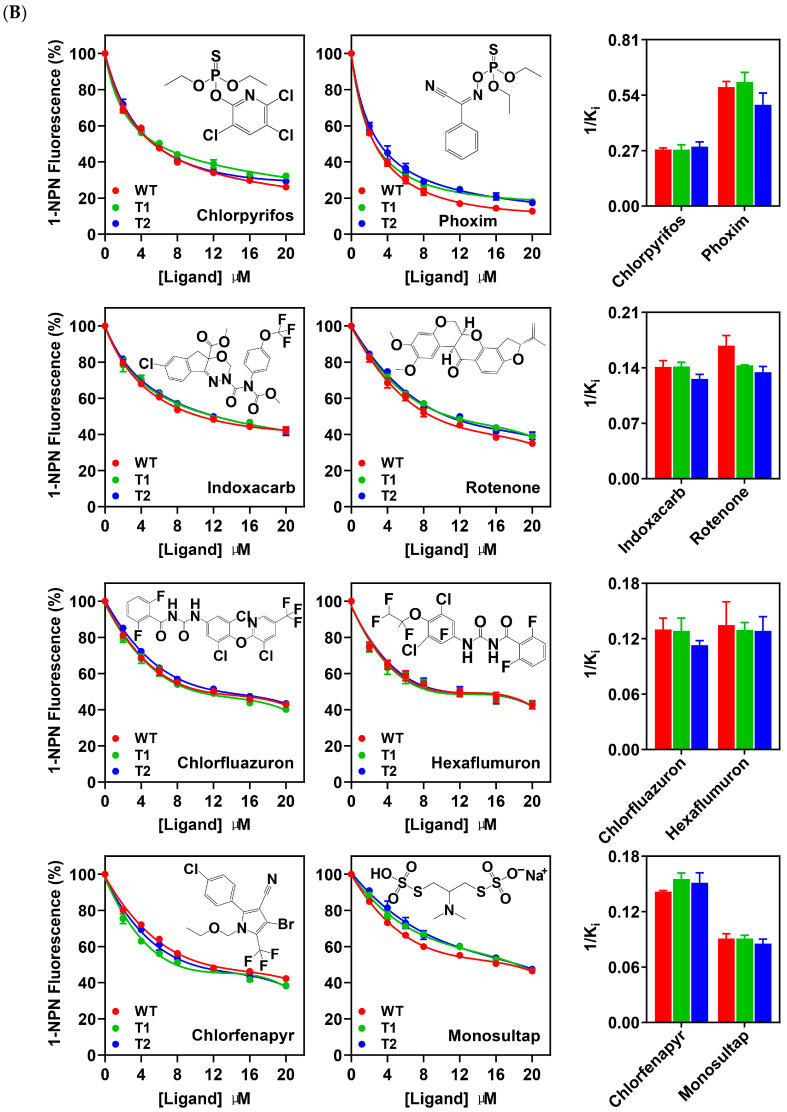
Comparison of the binding abilities of the wildtype (WT) and two truncated (T1 and T2) PxutCSP19s in *P. xuthus* to ligands with high affinities. (**A**) Comparison of the binding affinities of the wildtype and two truncated PxutCSP19s to six host volatiles. (**B**) Comparison of the binding affinities of the wildtype and two truncated PxutCSP19s to eight insecticides. The structures of the compounds were indicated. The reciprocals of the K_i_ values between the wildtype and two truncated PxutCSP19s were compared by Student’s *t*-test (*p* < 0.05). There were no significant differences in all the pairwise comparisons.

To test the effects of the N-terminal sequences on the binding of PxutCSP19 to ligands, six host odorants and eight insecticides showing high affinities were selected. As a result, the two truncated PxutCSP19s retained the high affinities with host volatiles and insecticides, as very similar IC_50_ and K_i_ values were observed between the wildtype and the truncated proteins (*p* > 0.05). Moreover, the three PxutCSP19 proteins had almost the same binding abilities at each concentration of one ligand (Figure 5A,B and Table 2). Thus, our study experimentally evidenced that this extended N-terminal region of PxutCSP19 did not significantly affect its binding abilities with ligands.

## 4. Discussion

The butterfly constitutes an important phylogenetic clade of the Lepidoptera, as a typical representative of herbivorous insects. In comparison to moths, butterfly CSPs have received less attention with respect to their roles in the perception of host odorants, particularly for sex-biased CSPs that guide female- or male-specific olfactory behaviors. The swallowtail butterfly *P. xuthus* provides an excellent model for investigating insect–host interactions where larvae feed solely on plant species of the Rutaceae family and adults are important flower-visiting insects [4,5]. This olfactory system is of paramount importance for larval feeding and female oviposition in this butterfly, where olfactory proteins enriched in antennae, including CSPs, mediate these life activities [39]. Here, we characterized a female-antenna-enriched PxutCSP19 in *P. xuthus* and its orthologs in other lepidopteran species, emphasizing the putative roles of PxutCSP19 in host recognition and insecticide resistance. Our findings have complemented the information on butterfly CSPs in olfaction and other functions.

When PxutCSP19 was identified from the transcriptome of *P. xuthus*, we noted that this protein possessed a particularly extended N-terminus (more than 35 amino acids compared to typical CSPs), as indicated in multiple sequence alignments and NCBI blast hits. We asked whether its orthologs in other lepidopteran insects also had this additional sequence at the N-terminal region. As expected, the PxutCSP19 orthologs in 22 other *Papilio* butterflies and five other lepidopteran species also exhibited such an extended N-terminus, suggesting the importance of this N-terminus segment in the interactions of proteins and ligands, possibly like the C-terminal regions of OBPs [48,49]. In the analyses of the sequence alignments, the exon-1 sequences (88.46% identity) of *Papilio* CSP19s shared higher conservation than exon-2 (82.00% identity). Focusing on the exon-2 region, highly divergent loop 5 and helix α6 were detected, possibly contributing to the functional differences of *Papilio* CSP19s. Our expression profiling analyses revealed the predominant expression of PxutCSP19 in the female antennae, consistent with the result that this gene originated from the antennal transcriptome of female *P. xuthus* (accession number: DRX276979). This female-antenna-biased feature of PxutCSP19 may be associated with specific behaviors of female butterflies like the seeking of oviposition sites and the detection of male sex pheromones, thereby enabling us to further explore its roles in odorant detection. Due to the unknown sex pheromones of male *P. xuthus*, here, we did not test the interactions of PxutCSP19 to male sex pheromones and mainly focused on the putative roles of PxutCSP19 in the detection of host volatiles. Apart from antennae, it was also detectable for the expression of PxutCSP19 in legs and non-chemosensory body parts. A previous study indicated that RhorCSPs highly expressed in the tarsi of female and male *Rhaphuma horsfieldi* were involved in the binding of insecticides [46]. In female or male reproductive systems, insect CSPs were key participants in mediating reproduction, such as SexiCSP3 in *Spodoptera exigua* [50], SlitCSP19 in *Spodoptera litura* [51], DabiCSP15 in *D. abietella* [11] and OcomCSP12 in *Ophraella communa* [52]. Thus, PxutCSP19 in the swallowtail butterfly, which showed a diverse tissue expression profile, may be endowed with other roles in addition to olfaction.

Insect CSPs enriched in the female antennae are potential molecular targets for the seeking of oviposition sites [21,24,25,26]. Considering the preferential expression of PxutCSP19 in the female antennae of *P. xuthus*, we characterized its roles in the recognition of host odorants. As evidenced in the binding assays, 6 of the tested 47 host volatiles were identified as the optimal ligands (K_i_ < 23 μM) that generally existed in the Rutaceae plants [42,43,44]. In particular, a monoterpenoid linalool that widely present in the Rutaceae family and plant flowers has been found to elicit the electrophysiological and behavioral responses of several *Papilio* butterflies, including *P. xuthus* [53,54]. In *P. polytes*, adults exhibited the strongest electrophysiological activity to a host odorant linalool released by *Citrus sinensis* cv. Navel. Further behavioral assays demonstrated that this butterfly could be attracted by linalool and that female insects preferred to lay eggs on cards containing linalool [55]. However, it is still unknown which olfactory proteins in these *Papilio* butterflies are involved in the sensing of linalool. Apart from that, linalool was also a critical olfactory modulator in moths, beetles, bees, bugs, mosquitos, thrips, aphids and flies [56]. More importantly, potential molecular targets for the detection of linalool have been found in some insects, including insect OBPs like SfruOBP7 in *Spodoptera frugiperda* [57] and DcitOBP7 in *Diaphorina citri* [58], as well as insect ORs like BminOR24 in *Bactrocera minax* [59], AlucOR47 in *A. lucorum* [60], DsuzOR69aA in *Drosophila suzukii* [61], and OR29 and OR53 in *Anopheles gambiae* and *Anopheles stephensi* [62]. Our current study revealed that this female-antenna-biased PxutCSP19 could also interact strongly with linalool, suggesting its roles in the perception of linalool responsible for the seeking of ovipositing hosts, coupled with linalool-mediating olfactory behaviors of *P. xuthus* and its abundant presence in the Rutaceae [44,45,53].

Insect CSPs have been found to participate in insecticide resistance, as evidenced in moths, aphids, beetles and mosquitos [27,46,63,64]. Yet, studies on butterfly CSP-mediating resistance have not been reported to date. Given the strong flight ability and flower-visiting activities of the swallowtail butterfly, it will contact a diverse range of habitats, offering more possibilities of exposure to toxic substances like insecticides. We thus also investigated the interactions of PxutCSP19 and insecticides. In the binding experiments, some insecticides that have been demonstrated to be strongly bound by OBPs or CSPs were included, such as phoxim and chlorpyrifos [11,65,66,67]. On the other hand, insecticides widely used for the control of lepidopteran pests were also tested, such as emamectin benzoate, chlorfenapyr and chlorantraniliprole [68]. Expectedly, two organophosphorus broad-spectrum insecticides, phoxim and chlorpyrifos, had the highest binding affinities, which is in agreement with early studies on moth OBPs and CSPs [11,67,69,70]. It appeared that insect OBPs or CSPs were easily able to accommodate the two chemical molecules, possibly suggesting an adaptation of insects to a toxic environment or the plasticity of binding pockets of soluble olfactory proteins. Apart from that, it was noted that five other insecticides, including a botanical chemical rotenone, were also able to displace a large number of 1-NPN molecules (K_i_ < 10 μM) from the binding pocket of PxutCSP19. The high affinities of insect CSPs to these five insecticides were also observed in DabiCSPs in *D. abietella* [11], RhorCSPs in *Rhaphuma horsfieldi* [46] and PxylCSP1 in *P. xylostella* [63]. As butterflies are considered to have evolved from moths [1,3], some CSPs in butterflies (e.g., PxutCSP19 in *P. xuthus*) are likely to retain ancestral functions. All in all, our study revealed, for the first time, the importance of butterfly CSPs in the binding of insecticides, possibly helping butterflies improve their resistance levels.

In comparison, the N- and C-terminal sequences (also including residues at the C-termini) of insect OBPs have captured growing attention, with an emphasis on the importance in the specificity of ligand recognition [49,71,72]. Yet, the roles of the N- or C-terminal segments of insect CSPs remain unexplored to date. The present study revealed an extended N-terminal sequence of PxutCSP19 and its orthologs, providing an excellent object for studying the effects of the N-terminus on the ligand binding. Regardless of the host volatiles and insecticides, deletion of the N-terminus of PxutCSP19 did not significantly affect the specificity of the protein–ligand binding. A possible explanation was that this extended N-terminal region did not form one or more additional α-helixes occupying the binding pocket of PxutCSP19, unlike the C-termini of OBPs [48,49]. Notably, the structural resolution of OBPs has evidenced that their extended N-termini were unstructured, including AgamOBP1 and AgamOBP47 in *A. gambiae* [73,74] and AmelASP1 in *Apis mellifera* [75]. Thereby, it was suggested that the extended N-terminal segments of PxutCSP19 or its orthologs were likely to be disordered and did not determine the ligand-binding specificity.

## 5. Conclusions

In moths, female species are endowed with some specific olfactory behaviors, such as the location of oviposition sites. In nature, they mainly utilize olfactory-related proteins enriched in antennae to finish oviposition activities by recognizing host-derived volatiles [22,23]. Yet, the putative roles of butterfly CSPs in female oviposition remain unknown. In this study, we identified a female-antenna-biased PxutCSP19 in *P. xuthus*, which shared high conservation with its orthologs in other *Papilio* butterflies and lepidopteran species. Compared with insects’ typical CSPs, PxutCSP19 and its orthologs possessed a particularly extended N-terminal segment, which did not significantly affect the binding abilities of PxutCSP19 to ligands with high affinities. The interactions of PxutCSP19 and host volatiles revealed its importance in the search for host plants, particularly for six host-derived compounds, including linalool, showing strong binding abilities. We also characterized the roles of PxutCSP19 in the sequestering of insecticides, revealing eight ligands with high affinities, including phoxim and chlorpyrifos. Given the wide expression feature of PxutCSP19 from larvae to adults, it is suggested that the binding of this protein to insecticides may be extremely deleterious for the swallowtail butterfly. Yet, the complex mechanisms of soluble olfactory proteins in insecticide resistance other than chemoreception remain unknown. Altogether, these findings suggest the putative roles of PxutCSP19 responsible for the binding and recognition of host volatiles and insecticides.

## Figures and Tables

**Table 1:** Information on the CSP19 orthologs in 23 *Papilio* butterflies.

Species	ORF(bp)/ Full-Length	Signal Peptide (aa)	Exon Length (bp)		Intron			Orientation	Chromosome	Note
			Exon-1	Exon-2	Number	Length (bp)	Phase			
*P. polytes*	471/Yes	17	294	165	1	498	1	+	DF823923.1	
*P. xuthus*	462/Yes	17	294	165	1	578	1	+	Scaffold 50	
*P. machaon*	462/Yes	17	294	165	1	388	1	–	Scaffold 4	
*P. memnon*	471/Yes	17	294	174	1	518	1	+	Scaffold 26	
*P. dardanus*	462/Yes	17	294	165	1	291	1	+	Scaffold 115	
*P. bianor*	462/Yes	17	294	165	1	671	1	–	TM66B3WUVR 8	
*P. aristodemus*	456/Yes	16	291	162	1	326	1	–	JZZY7UMRXG 8	
*P. glaucus*	462/Yes	16	288	171	1	792	1	–	scaffold_2208	
*P. clytia*	453/Yes	17	288	162	/	/	/	+	cov_5.555223 + cov_5.608469	
*P. antimachus*	462/Yes	17	294	165	1	502	1	–	cov_36.791104	
*P. slateri*	378/No	17	/	/	/	/	/	+	cov_5.088006	Missing ~25 amino acids in the middle of this gene
*P. alexanor*	462/Yes	17	294	165	1	629	1	–	cov_28.334434	
*P. ambrax*	471/Yes	17	294	174	1	446	1	–	cov_5.029448	
*P. phestus*	471/Yes	17	294	174	1	467	1	–	cov_3.748094	
*P. polyxenes*	462/Yes	17	294	174	1	498	1	+	Scaffold3300	
*P. zelicaon*	459/Yes	17	294	165	/	/	/	–	cov_68.359788 + cov_43.479663	
*P. protenor*	471/Yes	17	294	174	1	724	1	+	ctg7	
*P. gigon*	462/Yes	17	294	165	1	409	1	–	0KYF3WRZ5T 8	
*P. thoas*	456/Yes	16	291	162	/	/	/	/	cov_54.059850 + cov_21.006012 + cov_45.507022	
*P. joanae*	462/Yes	17	294	165	/	/	/	+	cov_10.450777 + cov_17.501926	
*P. demoleus*	462/Yes	17	294	165	1	358	1	+	ctg11	
*P. elwesi*	453/Yes	17	288	162	1	921	1	–	Chromosome 16	
*P. helenus*	402/No	17	/	/	/	/	/	–	MHAD71OSIT 15	Missing ~23 amino acids in the middle of this gene

Note: ORF means open reading frame. aa means amino acids. “/” means unknown information of genes.

**Table 2:** Binding affinities of a female-antenna-biased PxutCSP19 in *P. xuthus* to 47 host volatiles and 28 insecticides.

Compound	PxutCSP19								
	Wildtype (WT)			T1			T2		
	F.D. (%)	IC_50_ (μM)	K_i_ (μM)	F.D. (%)	IC_50_ (μM)	K_i_ (μM)	F.D. (%)	IC_50_ (μM)	K_i_ (μM)
Host volatiles									
Alkenes									
α-Pinene	34.65	>20	–						
(1R)-(+)-α-Pinene	38.38	>20	–						
β-Pinene	31.21	>20	–						
Myrcene	32.40	>20	–						
Farnesene	23.21	>20	–						
Ocimene	42.70	31.27 ± 1.73	20.61 ± 1.14	43.11	31.71 ± 1.86	19.93 ± 1.17	35.19	32.82 ± 1.09	21.61 ± 0.72
3-Carene	35.12	>20	–						
α-Caryophyllene	7.48	>20	–						
β-Caryophyllene	NB		–						
Limonene	20.36	>20	–						
Sabinene	33.51	>20	–						
γ-Terpinene	46.40	>36	–						
α-Terpinene	40.75	>36	–						
α-Phellandrene	34.09	>20	–						
Camphene	34.52	>20	–						
Terpinolene	44.05	34.11 ± 1.31	22.48 ± 0.87	40.12	33.64 ± 0.16	21.14 ± 0.10	38.49	31.57 ± 1.67	20.78 ± 1.10
β-Elemene	NB		–						
*p*-Isopropyl toluene	32.32	>20	–						
Alcohols									
1-Hexanol	38.95	>20	–						
(E)-2-Hexen-1-ol	35.63	>20	–						
(Z)-3-Hexen-1-ol	35.80	>20	–						
1-Octanol	42.92	34.46 ± 1.11	22.71 ± 0.73	43.54	34.48 ± 0.70	21.67 ± 0.44	42.67	34.61 ± 0.64	22.79 ± 0.42
Phenethyl alcohol	39.66	>20	–						
Nerolidol	12.36	>20	–						
Farnesol	27.04	>20	–						
Linalool	45.65	33.84 ± 0.84	22.30 ± 0.55	39.75	33.14 ± 1.34	20.83 ± 0.85	40.26	34.58 ± 0.90	22.77 ± 0.59
Geraniol	39.03		–						
Nerol	44.09	33.99 ± 1.46	22.40 ± 0.96	39.39	34.75 ± 0.03	21.84 ± 0.02	40.79	33.78 ± 0.85	22.24 ± 0.56
4-Terpineol	45.13	>36	–						
α-Terpineol	41.46	>36	–						
Aldehydes									
Hexanal	21.08	>20	–						
(E)-2-Hexenal	33.76	>20	–						
Octanal	22.75	>20	–						
Nonanal	29.30	>20	–						
Decanal	28.74	>20	–						
Undecanal	27.70	>20	–						
Benzaldehyde	36.91	>20	–						
Phenylacetaldehyde	27.32	>20	–						
Syringaldehyde	43.39	31.02 ± 0.97	20.44 ± 0.64	42.68	28.09 ± 1.33	17.66 ± 0.84	42.18	28.58 ± 1.12	18.82 ± 0.74
Citronellal	34.20	>20	–						
Citral	39.68	>20	–						
Esters									
Ethyl butyrate	28.67	>20	–						
Ethyl acetate	37.65	>20	–						
Geranyl acetate	33.33	>20	–						
Others									
Methyl o-toluate	37.28	>20	–						
Indole	39.05	>20	–						
Carvacrol	37.40	>20	–						
Synthetic insecticides									
Benzoylureas									
Chlorbenzuron	42.75	>20	–						
Chlorfluazuron	60.51	11.88 ± 1.04	7.83 ± 0.68	54.70	12.72 ± 1.49	7.99 ± 0.94	57.01	13.49 ± 0.60	8.88 ± 0.40
Diflubenzuron	26.96	>20	–						
Hexaflumuron	59.55	12.15 ± 2.36	8.01 ± 1.56	56.46	12.37 ± 0.73	7.78 ± 0.46	59.61	12.18 ± 1.56	8.02 ± 1.03
Triflumuron	27.99	>20	–						
Pyrethroids									
α-Cypermethrin	5.65	>20	–						
Deltamethrin	39.31	>20	–						
Diamide									
Chlorantraniliprole	43.56	>20	–						
Organophosphates									
Acephate	37.07	>20	–						
Chlorpyrifos	75.06	5.52 ± 0.16	3.64 ± 0.11	70.28	5.87 ± 0.47	3.69 ± 0.29	73.23	5.32 ± 0.40	3.51 ± 0.27
Phoxim	87.27	2.63 ± 0.12	1.73 ± 0.08	83.13	2.66 ± 0.20	1.67 ± 0.13	84.75	3.16 ± 0.35	2.08 ± 0.23
Profenofos	41.04	>20	–						
Trichlorphon	33.61	>20	–						
Pyrazole									
Fipronil	37.47	>20	–						
Chloronicotinyls									
Acetamiprid	42.89	>20	–						
Imidacloprid	41.72	>20	–						
Thiamethoxam	44.56	>20	–						
Carbamates									
Thiodicarb	37.39	>20	–						
Methomyl	45.40	>20	–						
Indoxacarb	61.54	10.83 ± 0.65	7.14 ± 0.43	59.12	11.27 ± 0.41	7.08 ± 0.26	62.53	12.11 ± 0.56	7.98 ± 0.37
Nereistoxin									
Monosultap	53.60	16.80 ± 1.01	11.07 ± 0.66	54.78	17.58 ± 0.73	11.05 ± 0.46	61.01	17.88 ± 1.02	11.77 ± 0.67
Ecdysome agonist									
Tebufenozide	48.54	>20	–						
Pyrrole									
Chlorfenapyr	58.72	10.71 ± 0.09	7.06 ± 0.06	63.82	10.29 ± 0.43	6.47 ± 0.27	64.33	10.14 ± 0.68	6.68 ± 0.45
Antibiotic									
Emamectin benzoate	46.98	>20	–						
Biopesticides									
Rotenone	67.63	9.15 ± 0.76	6.03 ± 0.50	61.84	11.12 ± 0.04	6.99 ± 0.03	64.39	11.35 ± 0.58	7.43 ± 0.39
Matrine	39.95	>20	–						
Azadirachtin	34.50	>20	–						
Rhodojaponin III	38.52	>20	–						

Note: NB represents no binding of PxutCSP19 to ligands. “–” represents the fact that the IC_50_ values of PxutCSP19 to ligands could not be calculated at the concentrations of 20 μM or 36 μM and their relative K_i_ values could not be detected. F.D. represents the fluorescent displacement rate of ligands at 20 μM. In the interactions of two truncated PxutCSP19s with ligands, six host volatiles and eight insecticides with high affinities were tested. The remaining 61 compounds were not measured in the binding assays of two truncated PxutCSP19s and thus their F.D. and K_i_ values are blank.

## Data Availability

The data will be available on request.

## Supplementary Materials

The following supporting information can be downloaded at https://www.mdpi.com/article/10.3390/insects15070501/s1, Table S1. Information on the genomes in 27 lepidopteran species; Table S2. Primers used for the expression profiles and the prokaryotic expression of PxutCSP19 and its truncated proteins in *P. xuthus*; Supplementary File S1. Original gel images from the RT–PCR analyses in Figure 2; Supplementary File S2. Original gel images from the SDS-PAGE analyses in Figure 3; Figure S1. Binding properties of PxutCSP19 in *P. xuthus* to 1-NPN and ligands. (A) Binding of PxutCSP19 to the fluorescent probe and a relative Scatchard plot. The structure of the 1-NPN molecule was inserted. (B) Competitive binding curves of PxutCSP19 to different host volatiles and insecticides. All the tested compounds were plotted, including 45 host volatiles and 28 insecticides, except for β-caryophyllene and β-elemene, which had no binding with PxutCSP19.
