# Supplementary material for: A Female-Biased Chemosensory Protein PxutCSP19 in the Antennae of Papilio xuthus Tuned to Host Volatiles and Insecticides
Source: Insects. 2024 Jul 5;15(7):501. doi: 10.3390/insects15070501 (PMC11276849; doi:10.3390/insects15070501)
Supplement: Supplementary file 1 [file insects-15-00501-s001.zip › Figure S1.pdf]

Figure S1

A

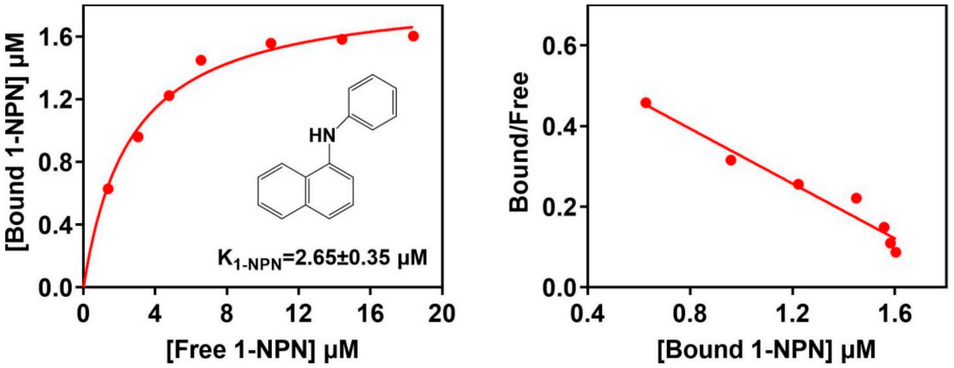
[Bound 1-NPN] μM
[Free 1-NPN] μM
HN
K1-NPN=2.65±0.35 μM
Bound/Free
[Bound 1-NPN] μM

B

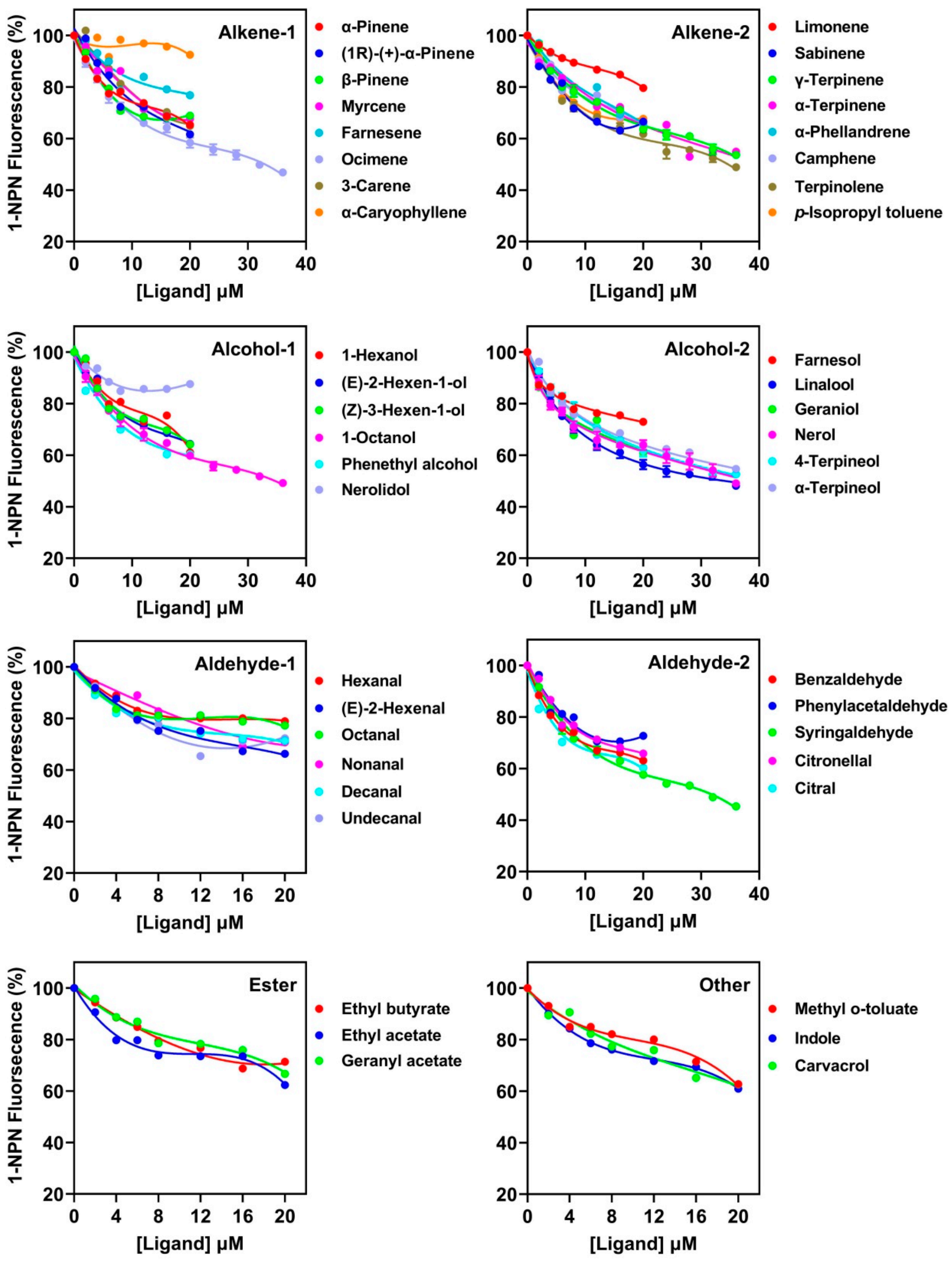
1-NPN Fluorescence (%)
[Ligand] μM
Alkene-1
α-Pinene
(1R)-(+)-α-Pinene
β-Pinene
Myrcene
Farnesene
Ocimene
3-Carene
α-Caryophyllene
Alkene-2
Limonene
Sabinene
γ-Terpinene
α-Terpinene
α-Phellandrene
Camphene
Terpinolene
p-Isopropyl toluene
Alcohol-1
1-Hexanol
(E)-2-Hexen-1-ol
(Z)-3-Hexen-1-ol
1-Octanol
Phenethyl alcohol
Nerolidol
Alcohol-2
Farnesol
Linalool
Geraniol
Nerol
4-Terpineol
α-Terpineol
Aldehyde-1
Hexanal
(E)-2-Hexenal
Octanal
Nonanal
Decanal
Undecanal
Aldehyde-2
Benzaldehyde
Phenylacetaldehyde
Syringaldehyde
Citronellal
Citral
1-NPN Fluorsecence (%)
Ester
Ethyl butyrate
Ethyl acetate
Geranyl acetate
Other
Methyl o-toluate
Indole
Carvacrol

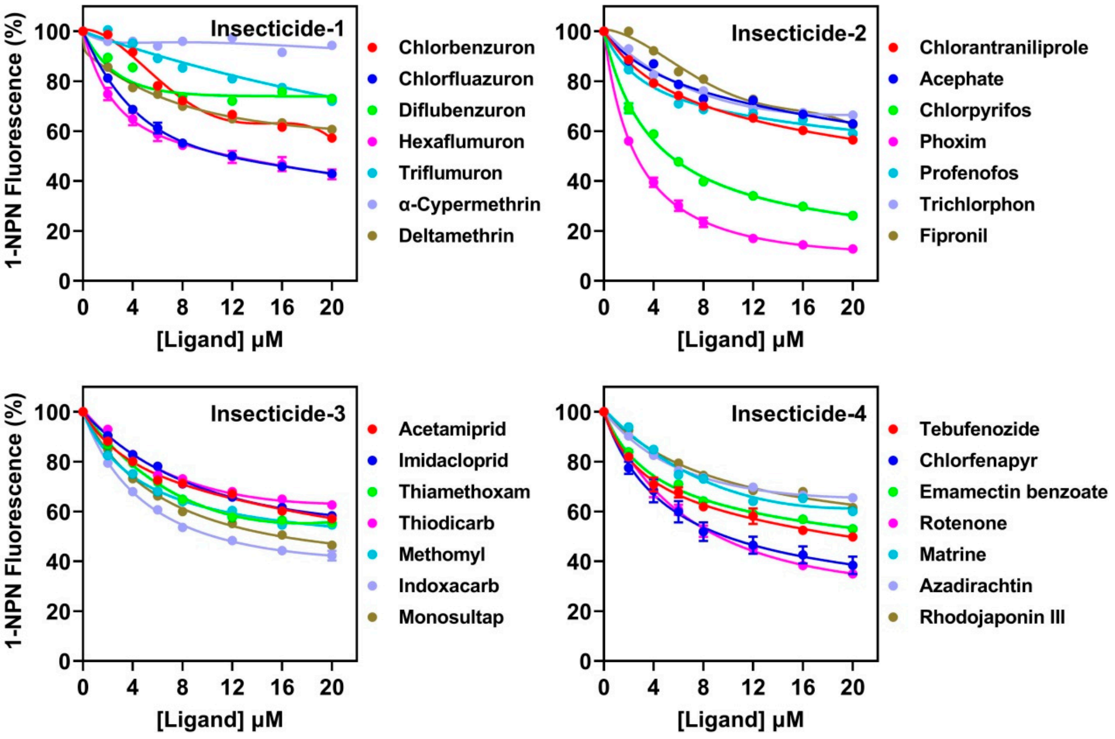
Insecticide-1
Chlorbenzuron
Chlorfluazuron
Diflubenzuron
Hexaflumuron
Triflumuron
α-Cypermethrin
Deltamethrin
Insecticide-2
Chlorantraniliprole
Acephate
Chlorpyrifos
Phoxim
Profenofos
Trichlorphon
Fipronil
Insecticide-3
Acetamiprid
Imidacloprid
Thiamethoxam
Thiodicarb
Methomyl
Indoxacarb
Monosultap
Insecticide-4
Tebufenozide
Chlorfenapyr
Emamectin benzoate
Rotenone
Matrine
Azadirachtin
Rhodojaponin III
1-NPN Fluorescence (%)
[Ligand] μM
