# Supplementary material for: A Female-Biased Chemosensory Protein PxutCSP19 in the Antennae of Papilio xuthus Tuned to Host Volatiles and Insecticides
Source: Insects. 2024 Jul 5;15(7):501. doi: 10.3390/insects15070501 (PMC11276849; doi:10.3390/insects15070501)
Supplement: Supplementary file 1 [file insects-15-00501-s001.zip › Supplementary file 1-Original gel images for Figure 2A.pdf]

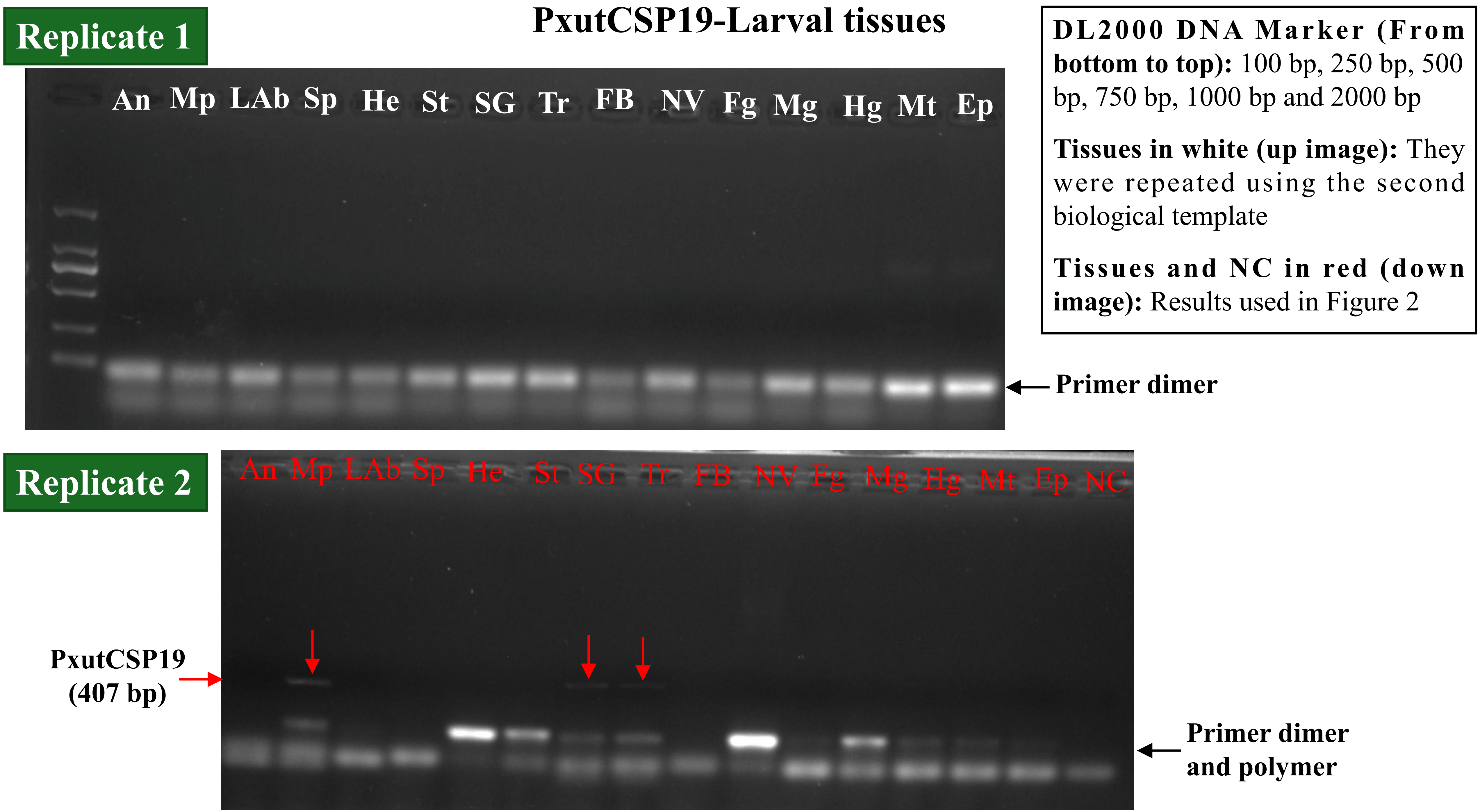

PxutCSP19-Larval tissues
Replicate 1
An Mp LAb Sp He St SG Tr FB NV Fg Mg Hg Mt Ep
Primer dimer
Replicate 2
An Mp LAb Sp He St SG Tr FB NV Fg Mg Hg Mt Ep NC
PxutCSP19 (407 bp)
Primer dimer and polymer
DL2000 DNA Marker (From bottom to top): 100 bp, 250 bp, 500 bp, 750 bp, 1000 bp and 2000 bp
Tissues in white (up image): They were repeated using the second biological template
Tissues and NC in red (down image): Results used in Figure 2

Replicate 1

PxutRPS4-Larval tissues

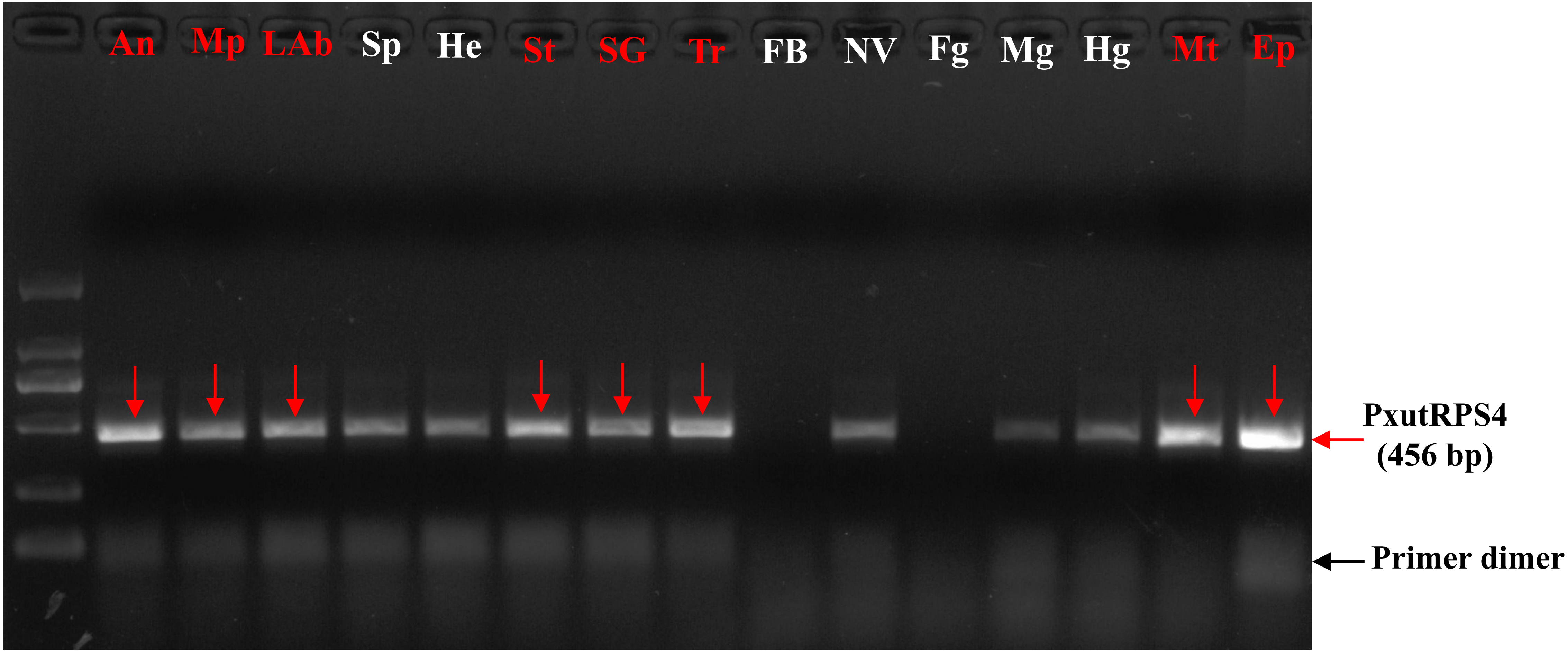

DL2000 DNA Marker (From bottom to top): 100 bp, 250 bp, 500 bp, 750 bp, 1000 bp and 2000 bp

Tissues in red: Results used in Figure 2

All 15 tissues were repeated using the second biological template

Red arrows: Target bands of PxutCSP19

Replicate 2

PxutRPS4-Larval tissues

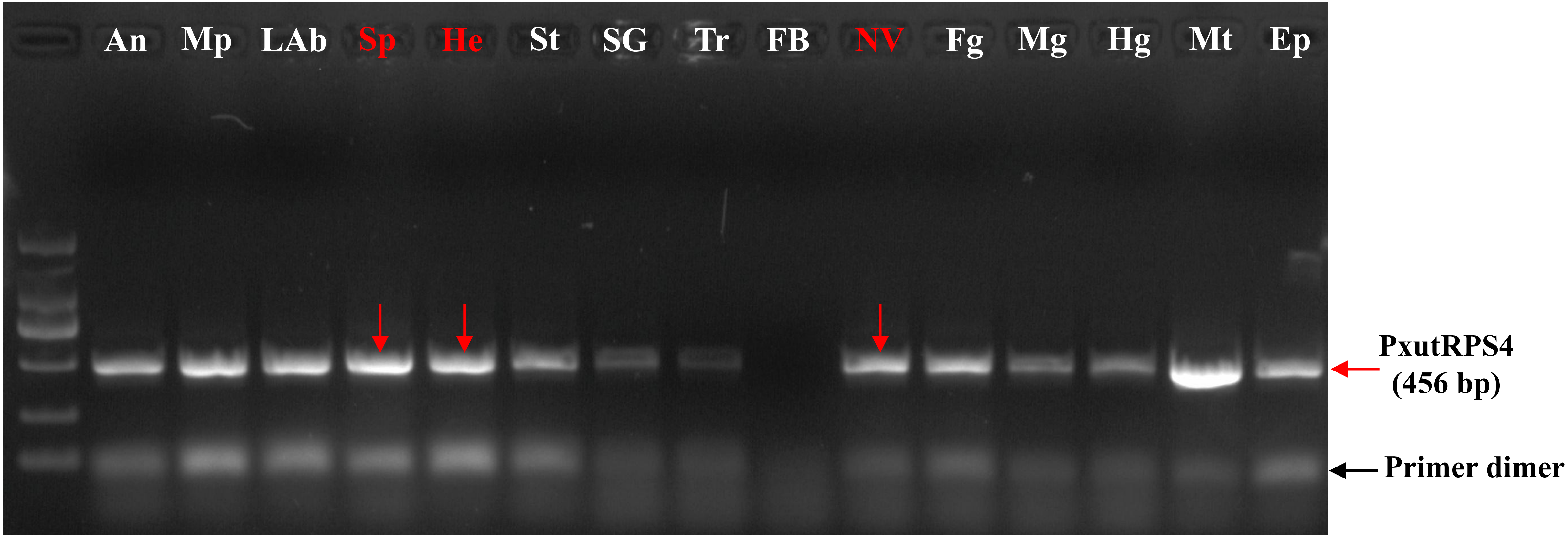

DL2000 DNA Marker (From bottom to top): 100 bp, 250 bp, 500 bp, 750 bp, 1000 bp and 2000 bp

Tissues in red: Results used in Figure 2

Four tissues (FB, Fg, Mg and Hg) were repeated using the third biological template

Red arrows: Target bands of PxutCSP19

Replicate 3

PxutRPS4-Larval tissues

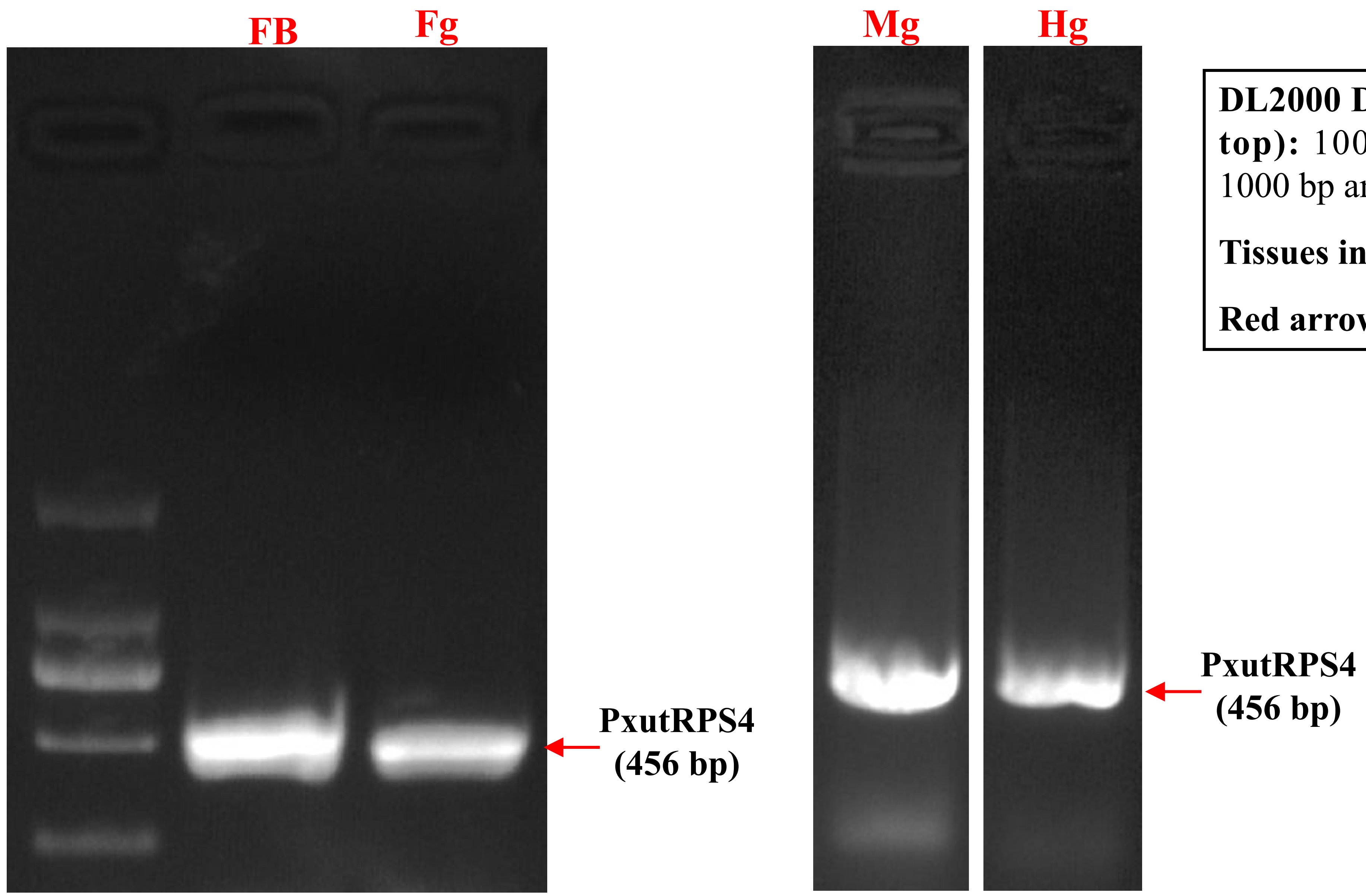

DL2000 DNA Marker (From bottom to top): 100 bp, 250 bp, 500 bp, 750 bp, 1000 bp and 2000 bp

Tissues in red: Results used in Figure 2

Red arrows: Target bands of PxutCSP19

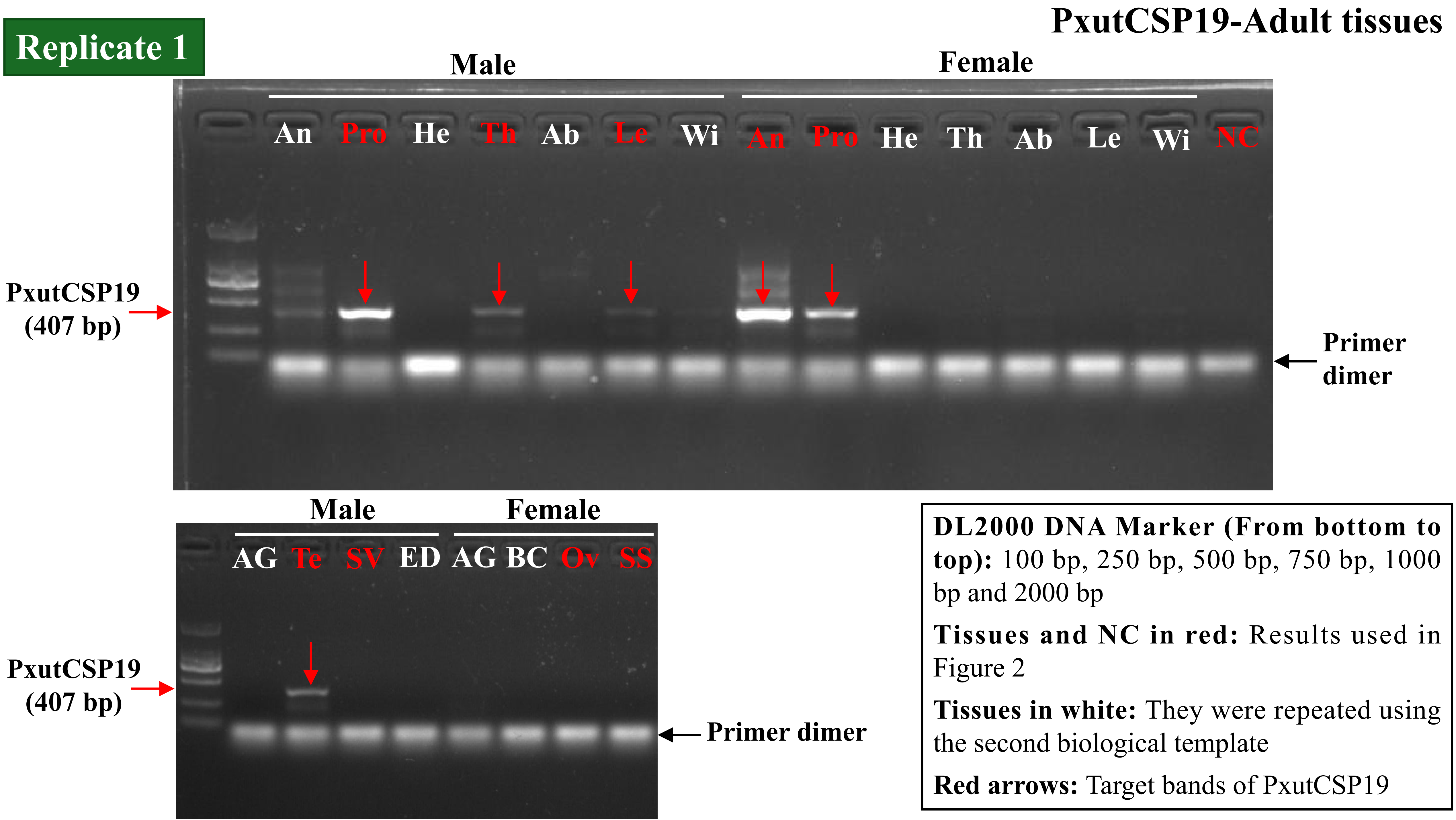

Replicate 1
PxutCSP19-Adult tissues
Male
Female
An Pro He Th Ab Le Wi An Pro He Th Ab Le Wi NC
PxutCSP19
(407 bp)
Primer
dimer
Male
Female
AG Te SV ED AG BC Ov SS
PxutCSP19
(407 bp)
Primer dimer
DL2000 DNA Marker (From bottom to top): 100 bp, 250 bp, 500 bp, 750 bp, 1000 bp and 2000 bp
Tissues and NC in red: Results used in Figure 2
Tissues in white: They were repeated using the second biological template
Red arrows: Target bands of PxutCSP19

Replicate 2

PxutCSP19-Adult tissues

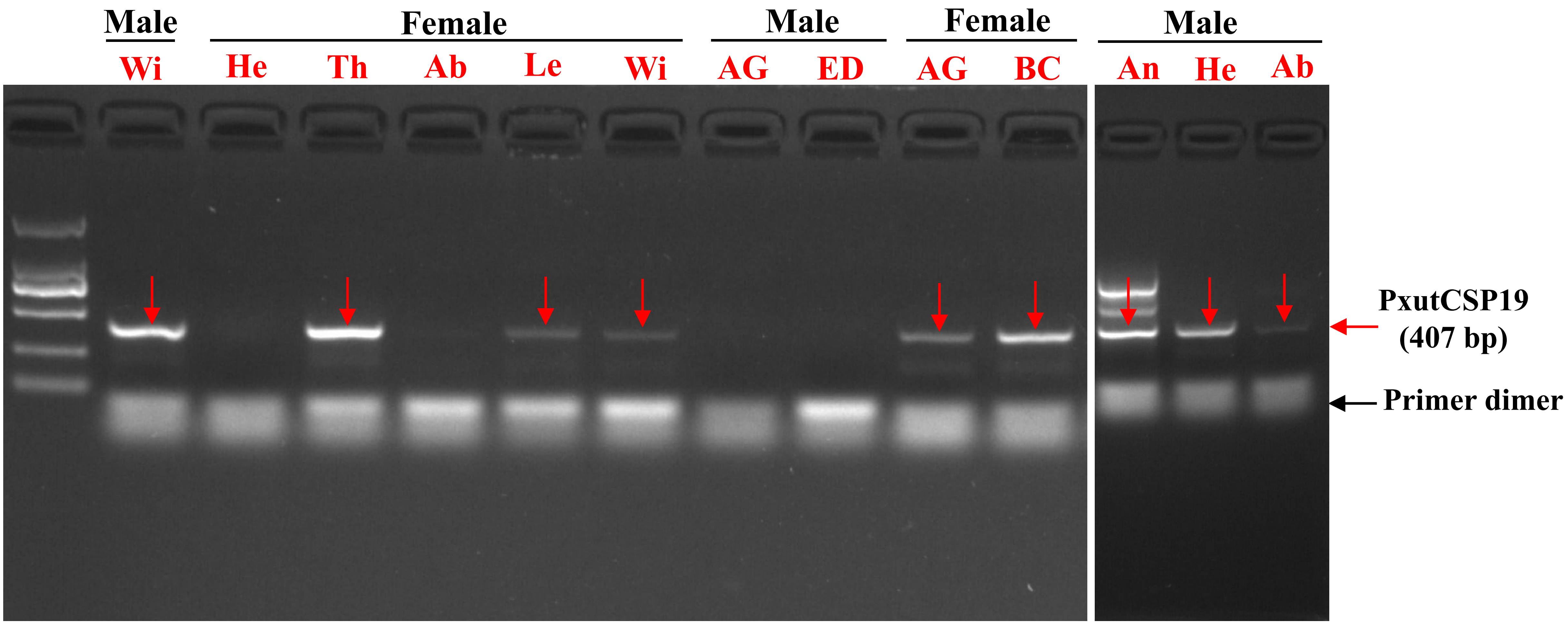

DL2000 DNA Marker (From bottom to top): 100 bp, 250 bp, 500 bp, 750 bp, 1000 bp and 2000 bp

Tissues in red: Results used in Figure 2.

Red arrows: Target bands of PxutCSP19

PxutRPS4-Adult tissues

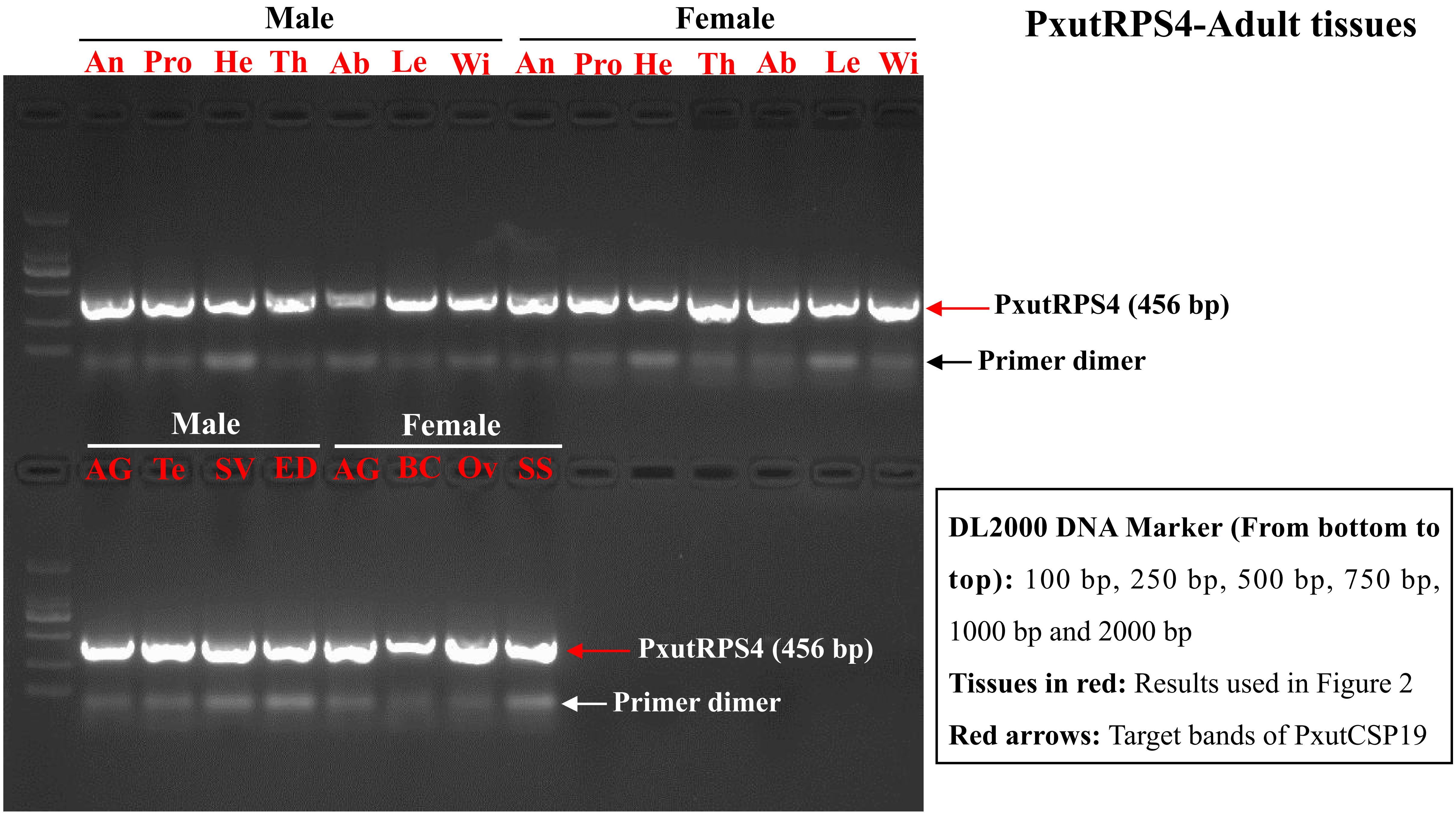

DL2000 DNA Marker (From bottom to top): 100 bp, 250 bp, 500 bp, 750 bp, 1000 bp and 2000 bp

Tissues in red: Results used in Figure 2

Red arrows: Target bands of PxutCSP19
