# Supplementary material for: A Female-Biased Chemosensory Protein PxutCSP19 in the Antennae of Papilio xuthus Tuned to Host Volatiles and Insecticides
Source: Insects. 2024 Jul 5;15(7):501. doi: 10.3390/insects15070501 (PMC11276849; doi:10.3390/insects15070501)
Supplement: Supplementary file 1 [file insects-15-00501-s001.zip › Supplementary file 2-Original gel images for Figure 3.pdf]

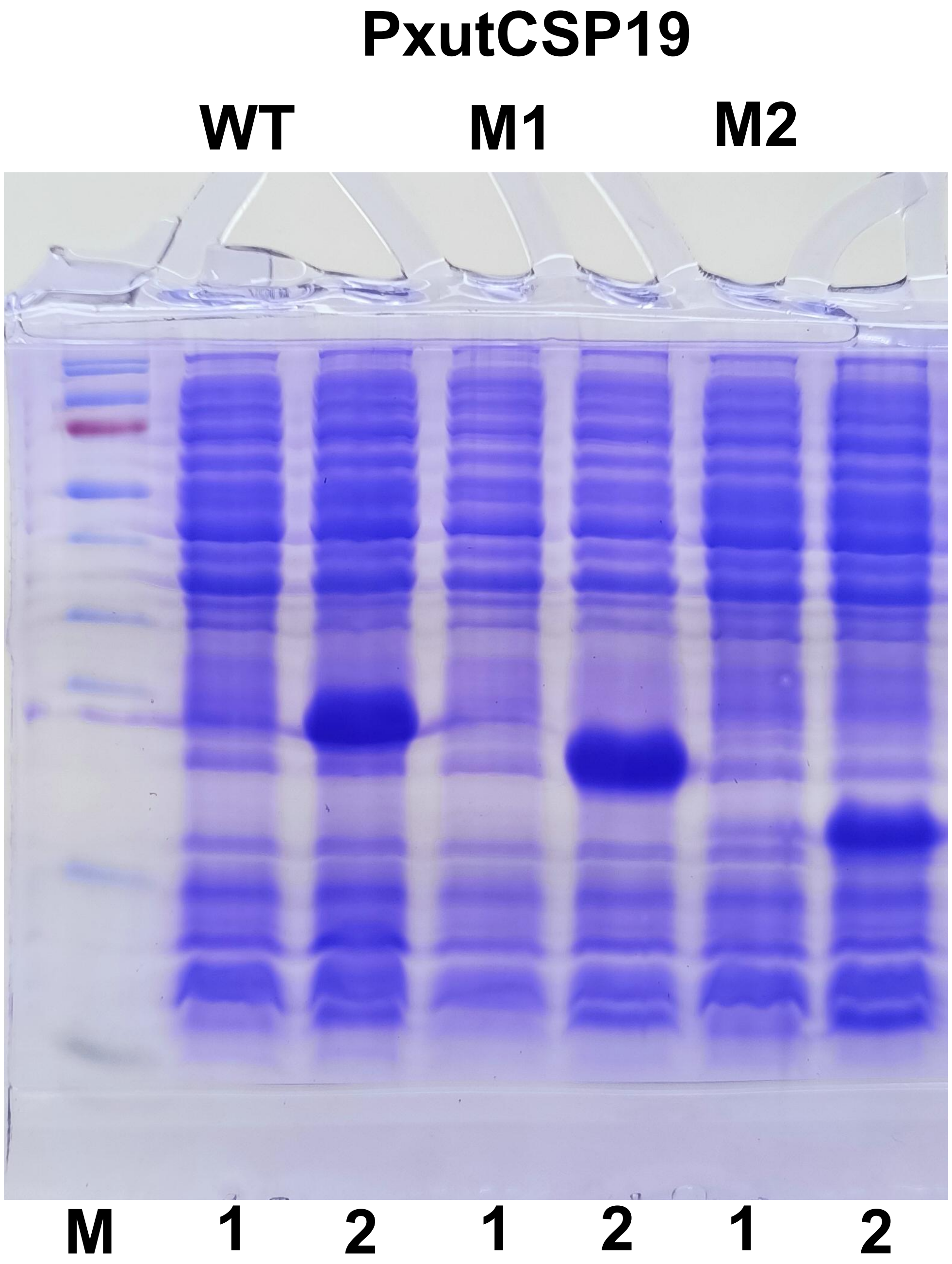

Protein molecular weight marker (From bottom to top): 10 kDa, 15 kDa, 25 kDa, 35 kDa, 40 kDa, 55 kDa, 70 kDa, 100 kDa, 130 kDa and 180 kDa.

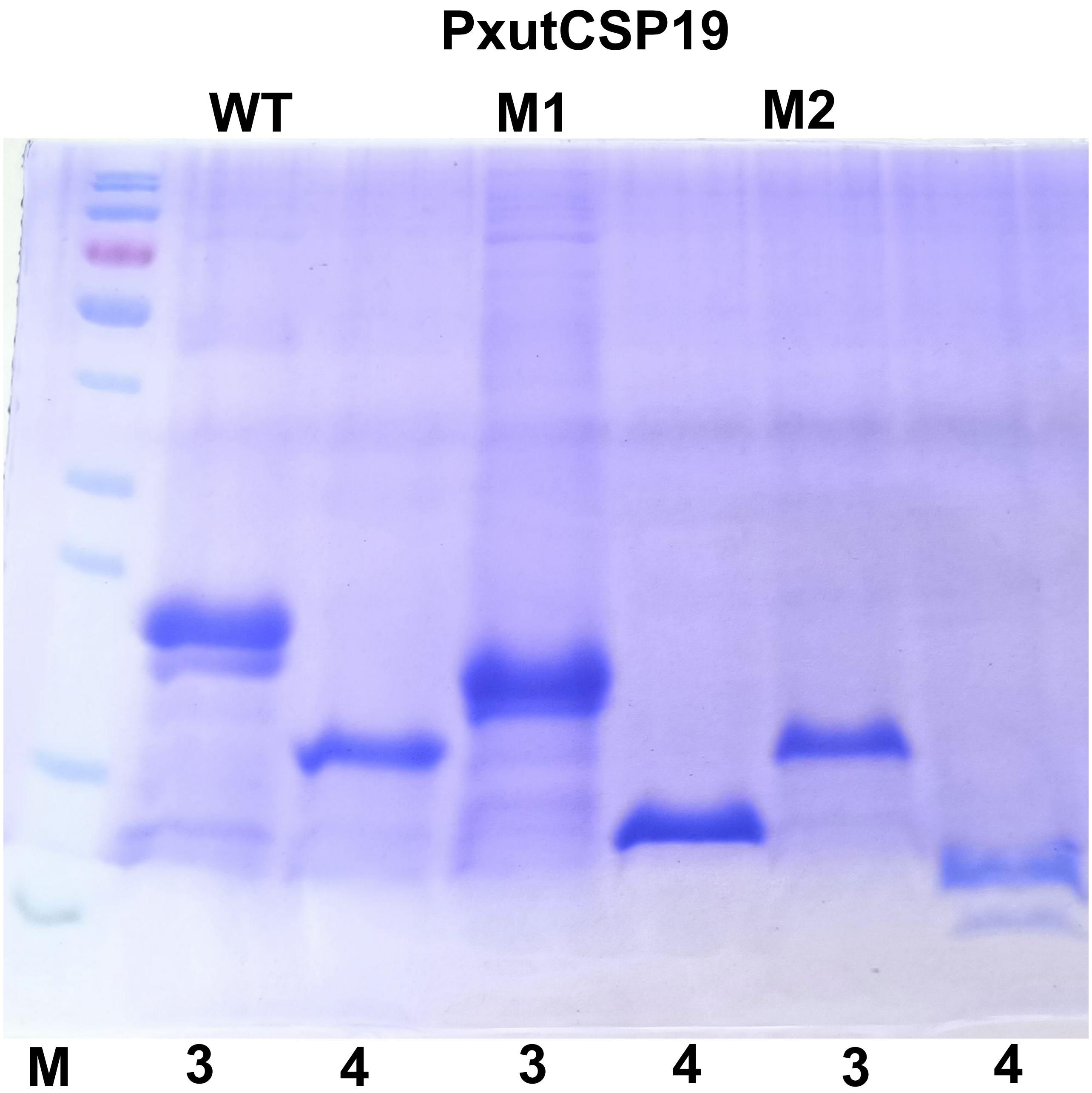
PxutCSP19
WT
M1
M2
M
3
4
3
4
3
4
