# Supplementary material for: A Female-Biased Chemosensory Protein PxutCSP19 in the Antennae of Papilio xuthus Tuned to Host Volatiles and Insecticides
Source: Insects. 2024 Jul 5;15(7):501. doi: 10.3390/insects15070501 (PMC11276849; doi:10.3390/insects15070501)
Supplement: Supplementary file 1 [file insects-15-00501-s001.zip › Table S1.pdf]

Table S1. Information on genomes in 27 lepidopteran species.

Species	Accession number	Version	Release date
Papilio polytes	GCA_000836215.1	Ppol_1.0	Feb 2, 2015
Papilio xuthus	GCA_000836235.2	Pxut_1.1	Feb 2, 2015
Papilio machaon	GCF_912999745.1	ilPapMach1.1	Aug 18, 2021
Papilio memnon	GCA_003118335.3	Papilio_memnon_Mimetic1	Mar 2, 2018
Papilio dardanus	GCA_013186455.1	ASM1318645v1	May 28, 2020
Papilio bianor	GCA_011763625.2	ASM1176362v2	Nov 21, 2022
Papilio aristodemus	GCA_016277805.2	ASM1627780v2	Nov 30, 2022
Papilio glaucus	GCA_000931545.1	pgl_assembly_v1	Feb 23, 2015
Papilio clytia	GCA_018247735.1	SRR8548584	May 3, 2021
Papilio antimachus	GCA_018246315.1	SRR8954523	May 3, 2021
Papilio slateri	GCA_018246275.1	SRR8954521	May 3, 2021
Papilio alexanor	GCA_018246295.1	SRR8954526	May 3, 2021
Papilio ambrax	GCA_018231605.1	SRR5879306	May 3, 2021
Papilio phestus	GCA_018231625.1	SRR5879277	May 3, 2021
Papilio polyxenes	GCA_026167825.1	ASM2616782v1	Nov 14, 2022
Papilio zelicaon	GCA_018246175.1	SRR8954529	May 3, 2021
Papilio protenor	GCA_029286645.1	kiz_Ppr_v1	Mar 20, 2023
Papilio gigon	GCA_027563995.1	ASM2756399v1	Jan 3, 2023
Papilio thoas	GCA_018246335.1	SRR8954522	May 3, 2021
Papilio joanae	GCA_018248035.1	SRR8954524	May 3, 2021
Papilio demoleus	GCA_029286655.1	kiz_Pde_v1	Mar 20, 2023
Papilio elwesi	GCA_029849275.1	ASM2984927v1	Apr 21, 2023
Papilio helenus	GCA_026546675.1	ASM2654667v1	Nov 30, 2022
Kallima inachus	https://doi.org/10.5061/dryad.8w9ghx3gt	Kin_Hic	May 18, 2020
Heliconius melpomene	GCA_013420985.1	ASM1342098v1	Jul 16, 2020
Manduca sexta	GCA_014839805.1	JHU_Msex_v1.0	Oct 2, 2020
Helicoverpa armigera	GCA_002156985.1	Harm_1.0	May 22, 2017
