# Supplementary material for: A Female-Biased Chemosensory Protein PxutCSP19 in the Antennae of Papilio xuthus Tuned to Host Volatiles and Insecticides
Source: Insects. 2024 Jul 5;15(7):501. doi: 10.3390/insects15070501 (PMC11276849; doi:10.3390/insects15070501)
Supplement: Supplementary file 1 [file insects-15-00501-s001.zip › Table S2.pdf]

Table S2. Primers used for expression profiles and the prokaryotic expression of PxutCSP19 and its truncated proteins in *P. xuthus*.

Gene	Primer sequence (5'-3')	Enzyme site
**RT–PCR analysis**		
CSP19-F	TTGTCAGGGACAGTTTTCTCTGA	–
CSP19-R	TGACGTTTGTTATACCTCGTAAGG	–
RPS4-F	AAGTTGACGGCAAAGTGAGGAC	–
RPS4-R	TCATCCTAGTGGCGAATGTGTG	–
**qPCR analysis**		
CSP19-qF	GTCAGGGACAGTTTTCTCT	–
CSP19-qR	CTTCTGCAATATCGTCTTCG	–
RPL8-F	AACCTGGAAGAGAAGATGG	–
RPL8-R	GCTTAACTCTAGTACGCTTG	–
**Prokaryotic expression**		
CSP19-eF	CGGGATCCGAAATAACAATAGGAGGAATTGAAAG	BamH I
CSP19-eR	CCGCTCGAGTTATCCTCGTTTCATGTCAATTATT	Xho I
**Two truncated PxutCSP19s**		
T1-F	CGGGATCCATGGGATATAAAATAATTTACGGC	BamH I
T1-R	CCGCTCGAGTTATCCTCGTTTCATGTCAATTATT	Xho I
T2-F	CGGGATCCGTTAAGAAAAAAGAGAAACTGGATATG	BamH I
T2-R	CCGCTCGAGTTATCCTCGTTTCATGTCAATTATT	Xho I

Note: The underlined bases represent restriction enzymes. F and R, forward and reverse primers, respectively.
